# Biofilm- and Spore-Disruptive Star-Shaped Poly(l‑lysine)/Hyaluronic Acid Microgels for Targeted Oral Therapy of *Clostridioides difficile* Infection

**DOI:** 10.1021/acs.biomac.5c02037

**Published:** 2026-04-22

**Authors:** I-Fan Lin, Chia-Yang Chou, Yu-Fang Chen, Ting-Yen Wang, Jing-Ting Lin, Ching-Chi Lee, Jenn-Wei Chen, Jeng-Shiung Jan, Yuan-Pin Hung

**Affiliations:** † Department of Microbiology and Immunology, College of Medicine, 38026 National Cheng Kung University, Tainan 70101, Taiwan; ‡ Division of Infectious Diseases, Department of Internal Medicine, E-Da Hospital, I-Shou University, Kaohsiung 82445, Taiwan; § School of Medicine, College of Medicine, I-Shou University, Kaohsiung 82445, Taiwan; ∥ Department of Chemical Engineering, 34912 National Cheng Kung University, Tainan 70101, Taiwan; ⊥ Department of Internal Medicine, College of Medicine, National Cheng Kung University Hospital, National Cheng Kung University, Tainan 70403, Taiwan; # Department of Medicine, College of Medicine, National Cheng Kung University, Tainan 70101, Taiwan; ∇ Program on Smart and Sustainable Manufacturing, Academy of Innovative Semiconductor and Sustainable Manufacturing, National Cheng Kung University, Tainan 70101, Taiwan; ○ Department of Internal Medicine, Tainan Hospital, Ministry of Health and Welfare, Tainan 70043, Taiwan

## Abstract

*Clostridioides difficile* infection (CDI) remains a major healthcare challenge due to recurrent disease, spore persistence, and biofilm-associated tolerance, while conventional antibiotics often disrupt gut microbiota. Here, we report a star-shaped poly­(l-lysine) dendrimer (G3-PLL_9_) formulated into hyaluronic acid–based microgels for targeted oral delivery to the inflamed colon. G3-PLL_9_ exhibited potent antimicrobial activity, including rapid bactericidal effects, superior spore inhibition compared with vancomycin, and robust biofilm disruption at subinhibitory concentrations. In a murine CDI model, rectal administration of G3-PLL_9_ alleviated clinical symptoms, reduced tissue damage, and lowered recurrence risk. To enable oral therapy, G3-PLL_9_ was incorporated into hyaluronic acid microgels, achieving site-specific release through hyaluronidase-mediated degradation in the inflamed colon. Importantly, treatment preserved commensal gut microbiota more effectively than vancomycin. Collectively, these findings highlight G3-PLL_9_ microgels as a microbiota-sparing therapeutic that targets multiple stages of CDI pathogenesis—including spores and biofilms—and demonstrate their potential for clinical translation.

## Graphical abstract

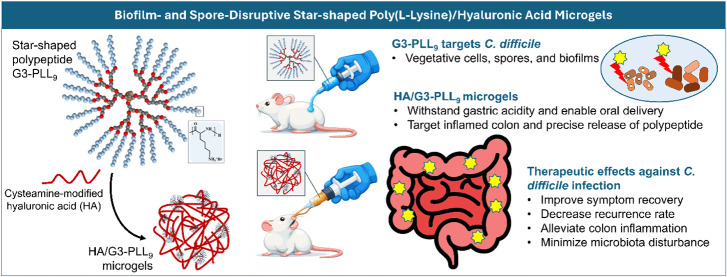

## 1 Introduction

*Clostridioides difficile* infection (CDI) remains a major cause of healthcare-associated gastrointestinal disease among hospitalized and antibiotic-exposed populations, particularly in developed regions such as North America, Europe, and East Asia.^1–3^ CDI is initiated by ingestion of spores that germinate into vegetative cells in the colon following disruption of normal gut microbiota.^2,4^ Notably, *C. difficile* spores exhibit exceptional resistance to conventional antibiotics and disinfectants, facilitating persistence in the host and contributing to recurrent CDI.^5–7^ The primary virulent factors of *C. difficile* are toxins A and B, encoded by the *tcdA* and *tcdB* genes, which damage colonic epithelial integrity, induce inflammation, and drive colitis.^2,8^ Additionally, certain hypervirulent *C. difficile* strains produce the binary toxin CDT, which has been implicated in enhanced bacterial adherence and colonization through the formation of microtubule-based protrusions on host epithelial cells.^8^ These virulence mechanisms, combined with spore resilience and biofilm-mediated tolerance, drive the high recurrence rates observed in CDI.^7–9^

Current therapeutic options for CDI are limited. Fidaxomicin is recommended as first-line therapy due to lower recurrence rates, while vancomycin remains an alternative treatment commonly prescribed, particularly in settings where fidaxomicin is not readily available or affordable.^2,10^ However, neither fidaxomicin nor vancomycin effectively eliminates spores or biofilms.^11–14^ Moreover, broad-spectrum antibiotic exposure, particularly vancomycin, can further disrupt the gut microbiome, increasing the risk of recurrence.^15,16^ These limitations underscore the need for novel antimicrobial strategies capable of targeting *C. difficile* vegetative cells, spores, and biofilms, while minimizing collateral damage to the intestinal microbiota.

Recent advances in polymer engineering have highlighted star-shaped polypeptides based on poly­(l-lysine) (PLL) as promising antimicrobial candidates.^17–20^ These polypeptides are synthesized via ring-opening polymerization (ROP) with biocompatible, hydrophilic, and noncytotoxic polyglycerol dendrimers (PGDs; Figure 1) as core initiators.^21–23^ Their unique star-shaped architecture integrates cationic and hydrophobic residues, resembling naturally occurring antimicrobial peptides and thereby enabling potent broad-spectrum antimicrobial activity.^17–19,24,25^ Studies have demonstrated the successful synthesis of polypeptides using initiators with primary and/or secondary hydroxyl groups,^26–28^ and our group has further developed star-shaped polypeptides with varying arm numbers, showing enhanced antimicrobial and spores- and biofilm-disrupting properties.^28^

**Figure 1:**
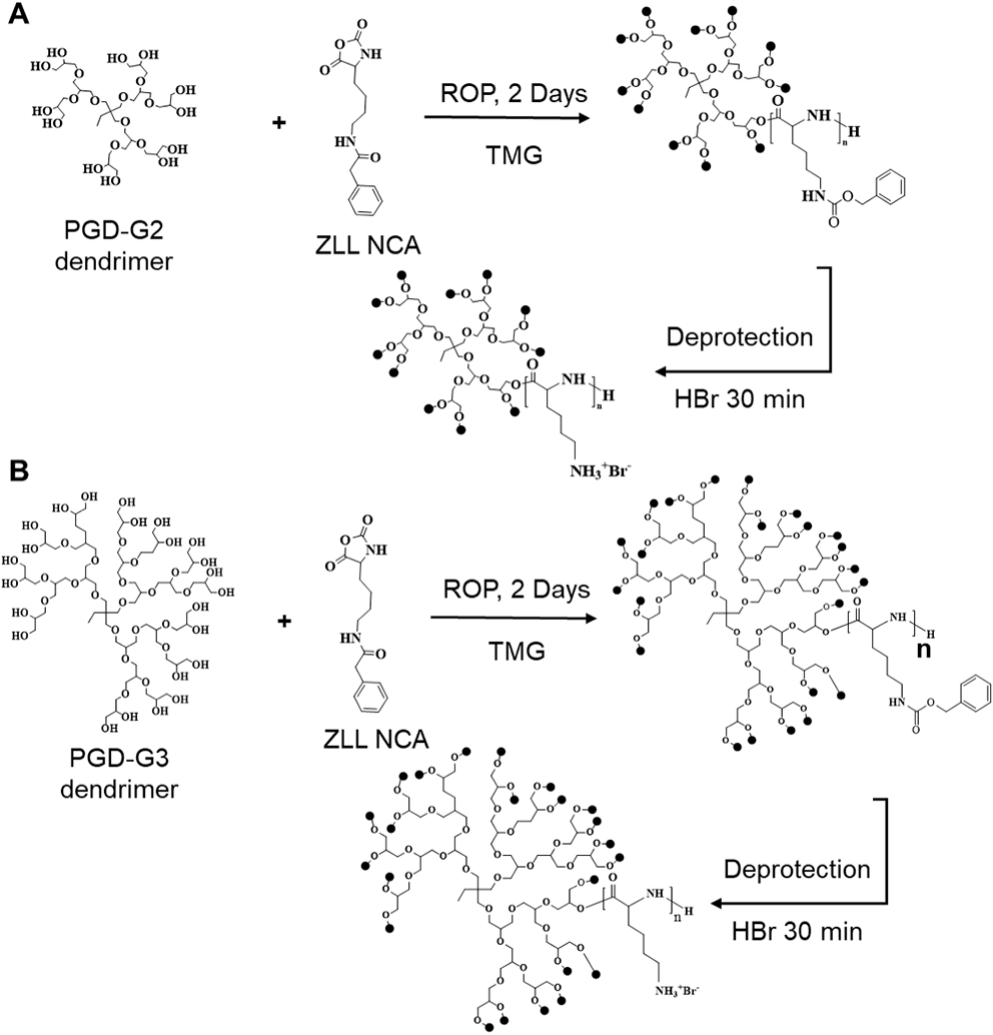
Synthesis of star-shaped polypeptides. Synthesis scheme of G2-PLL (A) and G3-PLL (B) using polyglycerol dendrimers (PGD) of generation 2 and 3 as core initiators, respectively. ZLL NCA, *Z*-l-lysine *N*-carboxyanhydride; ROP, ring-opening polymerization; TMG, 1,1,3,3-tetramethylguanidine; HBr, hydrogen bromide; NMR, nuclear magnetic resonance; GPC-LS, gel permeation chromatography-light scattering.

In this study, a positively charged 24-arm star-shaped PLL, G3-PLL_9_ (where the number denotes the chain length), was complexed with negatively charged hyaluronic acid (HA) via electrostatic interactions to form microgels. HA is a naturally occurring glycosaminoglycan with well-established biocompatibility and immunomodulatory properties.^29,30^ Complexation with HA improves the biocompatibility of G3-PLL_9_ and enables the HA/G3-PLL_9_ microgels to withstand gastric acidity and undergo hyaluronidase-mediated degradation in the inflamed colon, allowing localized payload release.^29,31–33^ This inflammation-responsive delivery minimizes off-target effects and enhances therapeutic precision,^30,34^ making HA attractive for oral drug delivery systems.^29,35^ By integrating these features, the constructed HA/G3-PLL_9_ microgels offer a promising inflamed-colon-targeted strategy capable of eradicating *C. difficile* vegetative cells along with its spores, and biofilms, key factors driving persistent colonization and CDI recurrence.^7,13,14,36–38^ This innovative approach addresses the limitations of current CDI treatments and represents a significant advancement in antimicrobial therapies.

## 2 Methods

### 2.1 Chemical Synthesis

#### 2.1.1 Synthesis and Characterization of Star-Shaped Polypeptides

Star-shaped poly­(l-lysine) polypeptides with 12 arms (G2-PLL) and 24 arms (G3-PLL) were synthesized via ROP using second- and third-generation PGD as core initiators (PGD-G2, *M*_n_ = 800 g mol^–1^; PGD-G3, *M*_n_ = 1689.8 g mol^–1^; Figure 1). PGDs were kindly synthesized by the Ooya group (Kobe University, Japan).^22^ Polymerization was initiated from hydroxyl termini activated with 1,1,3,3-tetramethylguanidine (TMG). The feed molar ratios of the initiator and ZLL NCA for synthesizing G2-PLL and G3-PLL were designated at 1:120 and 1:240, respectively. The degree of polymerization (DP) for the PLL segment was fixed at 10 per arm. The deprotection step involves removing the Z groups using a hydrogen bromide solution (Figure 1).^17,22^ The comprehensive synthetic procedures are detailed in the Supporting Information.

The star-shaped polypeptides were characterized by a proton nuclear magnetic resonance (^1^H NMR) spectroscopy and a gel permeation chromatography-light scattering (GPC-LS) system. ^1^H NMR analysis was conducted on a Bruker AVANCE III HD NMR (600 MHz) using TFA-*d*_1_ and D_2_O as the solvents. The GPC-LS analysis was conducted under the operating conditions at 55 °C and 0.8 mL min^–1^ of flow rate by using a Postnova PN3160 system equipped with a refractive index (RI) detector and a Postnova PN3609 MALS light scattering detector with nine angles. Two Shodex GPC columns (KD-802.5 and KD-804) were used for efficient separation with an eluent (*N*,*N*-dimethylformamide (DMF) solution containing 0.1 M lithium bromide) and a polystyrene standard (molecular weight: 277,000 g mol^–1^) for calibration. The G2-PZLL and G3-PZLL polypeptide samples, dissolved in DMF, were passed through a polytetrafluoroethylene Finetech filter (0.45 μm of pore size, 13 mm in diameter) before GPC-LS analysis.

#### 2.1.2 Preparation of HA/G3-PLL_9_ Microgels

To improve targeted delivery, G3-PLL_9_ was complexed with HA to form microgels. HA was modified with cysteamine (Cys) to introduce thiol groups via 1-ethyl-3-(3-(dimethylamino)­propyl) carbodiimide (EDC) and *N*-hydroxysuccinimide (NHS) activation of carboxyl groups as shown in Figure S1. Initially, HA (1 mmol) was dissolved in deionized water, followed by the addition of 1-ethyl-3-(3-(dimethylamino)­propyl) carbodiimide (EDC, 1.2 mmol) and *N*-hydroxysuccinimide (NHS, 2 mmol). The solution pH was adjusted to and maintained at 5.5 using 0.1 M HCl for 15 min to facilitate the activation of carboxyl groups. Subsequently, cysteamine (0.5 mmol) was added to the reaction mixture, and the reaction was allowed to proceed for 24 h. Upon completion of the reaction, the Cys-modified HA product was dialyzed against deionized water for 2 days at 4 °C to remove unreacted cysteamine, EDC, and NHS, followed by freeze-drying to obtain the purified Cys-modified HA with a yield of 90%. For the preparation of HA/G3-PLL_9_ microgels, the Cys-modified HA and G3-PLL_9_ solutions with the concentration of 2.0 mg mL^–1^ were separately prepared in deionized water and the G3-PLL_9_ solution was slowly added to the Cys-modified HA solution (10 mL) under ultrasonic conditions. The formation of colloidally stable HA/G3-PLL_9_ microgels was achieved by controlling the amount of G3-PLL_9_ solution added to the Cys-modified HA solution. Then 2.9 mL of G3-PLL_9_ solution was added to 10 mL of the Cys-modified HA solution without causing precipitation. For the resulting HA/G3-PLL_9_ microgel solution, the concentrations of G3-PLL_9_ and Cys-modified HA were 0.45 and 1.55 mg mL^–1^, respectively. The degree of substitution (DS) of Cys grafted on HA was characterized by NMR analysis. The hydrodynamic diameter (particle size), and zeta potential of the HA/G3-PLL_9_ microgels were measured using a dynamic light scattering (DLS) system (Otsuka ELSZ-1000). The measurements were conducted in triplicate (*n* = 3) and the autocorrelation functions in the data set were fitted by employing the cumulant method and CONTIN algorithms

### 2.2 Microbiological Assays

#### 2.2.1 Antimicrobial Activity of G3-PLL_9_

The toxigenic *C. difficile* strain JIK 8284 (tcdA^+^, tcdB^+^, ribotype 027), obtained from Professor Pei-Jane Tsai at National Cheng Kung University (NCKU), was used for antimicrobial evaluation, including determination of the minimum inhibitory concentration (MIC), minimum bactericidal concentration (MBC), and time-kill kinetics. Antimicrobial susceptibility testing was performed using a broth microdilution method, as agar-based assays are suboptimal for peptide-based antimicrobials and may be affected by heat during agar preparation. In brief, bacterial suspensions were standardized to 0.5 McFarland (1.5 × 10^8^ CFU mL^–1^) and diluted 100-fold in brain heart infusion supplement (BHIS) medium. G3-PLL_9_ concentrations were expressed in micromolar (μM) units due to its macromolecular polymeric nature, which facilitates mechanistic interpretation and ensures comparability across polymer batches. G3-PLL_9_ was 2-fold serially diluted and combined with bacterial suspensions in 3 mL test tubes. Controls included fidaxomicin, vancomycin, and BHIS alone, with *C. difficile* ATCC 700057 used as a quality-control strain in accordance with the Clinical and Laboratory Standards Institute (CLSI) guidelines.^39^ Following anaerobic incubation at 37 °C for 48 h, MIC was determined as the lowest concentration preventing visible growth. Because the intrinsic turbidity of G3-PLL_9_ solutions may interfere with visual MIC determination, the MBC values were further assessed by plating 100 μL aliquots from the MIC and higher concentrations onto CDC agar; *a* ≥99.9% colony reduction after 48 h indicated bactericidal activity. All procedures adhered to standardized microbiological protocols and were performed in triplicate using independent bacterial culture.^39,40^

#### 2.2.2 Sporicidal Assay

*C. difficile* spores (strain JIK 8284) were prepared and purified following established protocols (detailed in the Supporting Information).^41–43^ Spore formation was confirmed via malachite green staining under light microscopy, and spores were stored at 4 °C in the dark for three months. For sporicidal assessment, spores were subjected to sublethal heat treatment to reduce residual vegetative cells,^44^ exposed to G3-PLL_9_ for 30 min, and induced to germinate with taurocholic acid.^45^ Germination was quantified by plating serial dilutions on CDC agar and incubating anaerobically at 37 °C for 48 h prior to colony enumeration. Controls included nongermination, no treatment, fidaxomicin-treated, and vancomycin-treated spores. All experiments were performed in triplicate using independent spore preparations.

#### 2.2.3 Bacterial Biofilm Quantification

The effect of G3-PLL_9_ on *C. difficile* biofilm (strain JIK 8284) was evaluated by crystal violet staining.^46^ Biofilms were cultivated in 96-well plates by inoculating 200 μL of a 0.5 McFarland bacterial suspension diluted in BHIS and incubating anaerobically at 37 °C for 48 h.^36^ Wells were washed with phosphate-buffered saline (PBS), air-dried at 37 °C for 15 min, and treated with G3-PLL_9_ in triplicate for an additional 48 h. Biofilms were then dissolved in ethanol, stained with crystal violet, washed, and quantified by measuring OD_570_ using a microplate reader. Controls included blank wells, vehicle controls, no-treatment controls, and wells treated with fidaxomicin and vancomycin at their MICs. All experiments were conducted with three technical replicates and were independently repeated using separate biofilm preparations. The detailed procedure was in Supporting Information.

#### 2.2.4 Scanning Electron Microscopy

Scanning electron microscopy was performed to examine ultrastructural changes in *C. difficile* vegetative cells and spores (strain JIK 8284) following G3-PLL_9_ treatment. Overnight cultures of vegetative cells and purified spores were adjusted to an optical density at 600 nm (OD_600_) of 0.5 and treated with G3-PLL_9_ at final concentrations of 4–8 μM for 10, 30, or 60 min at room temperature. After treatment, samples were centrifuged at 6,000 rpm for 5 min, washed three times with PBS, and fixed in 2.5% (v/v) glutaraldehyde at 4 °C for 90 min. Fixed samples were washed again with PBS and applied as 10-μL droplets onto Formvar-coated silicon wafers, followed by air drying and vacuum drying for 30 min. Samples then underwent a secondary fixation with 2.5% glutaraldehyde on ice for 30 min, rinsed with PBS, and dehydrated through a graded ethanol series (30%, 50%, 70%, and 100%). Dehydrated specimens were subsequently treated with tert-butanol, stored at 4 °C to allow crystallization, and freeze-dried until complete sublimation. Finally, samples were sputter-coated with platinum and examined using a field-emission scanning electron microscope (SEM; JEOL JSM 6700F).

#### 2.2.5 Fluorescence Microscopy

Fluorescence microscopy assessed *C. difficile* spore (strain JIK 8284) viability after treatment with G3-PLL_9_ and staining using the Live/Dead BacLight Bacterial Viability Kit (Thermo Fisher Scientific). G3-PLL_9_ was labeled with an amine-reactive dye (Alexa Fluor 350, Thermo Fisher Scientific) to enhance visualization and specificity before cotreatment with *C. difficile* spores (detailed in the Supporting Information). Samples were examined with an upright fluorescent microscope (Olympus, BX53). Comparative studies included vancomycin and PBS controls. The proportion of viable and nonviable cells was calculated as the respective cell count divided by the total cell count (viable + nonviable) and expressed as a percentage.

### 2.3 Animal Models and Related Analyses

#### 2.3.1 CDI Mouse Model

The therapeutic efficacy of G3-PLL_9_ against CDI was evaluated using a previously established mouse model, modified from an earlier protocol.^43,47^ All animal experiments followed ethical guidelines and were approved by the Institutional Animal Care and Use Committee of NCKU (IACUC Approval No.: 112234). Housing conditions included a 13-h light/11-h dark cycle (7AM–8PM), temperatures of 23 ± 1 °C, and humidity levels of 45 ± 15%. After disruption of the gut microbiota using an antibiotic cocktail consisting of vancomycin (0.045 mg mL^–1^), metronidazole (0.215 mg mL^–1^), kanamycin (0.4 mg mL^–1^), colistin (0.057 mg mL^–1^), and gentamicin (0.035 mg mL^–1^), male C57BL/6JNral mice were infected with spores of *C. difficile* strain JIK 8284 (10^6^ spores per 100 μL). To prevent degradation in the stomach and duodenum, G3-PLL_9_ was administered via anal enema.^48^ Mice received 30-μL enemas of G3-PLL_9_ (0.25, 1, or 4 μM), fidaxomicin (30 mg kg^–1^ day^–1^) or vancomycin (50 mg kg^–1^ day^–1^) at 8, 24, and 48 h postinfection.^47^ HA/G3-PLL_9_ microgels, designed to facilitate targeted colon delivery, were administered orally at concentrations of 1, 2.5, and 5 μM in 100-μL volumes. The mock group was not infected with *C. difficile* and served as a control to assess microbiome changes following antibiotic pretreatment. Mice were monitored daily, and predefined humane end points (weight loss: A loss of 20% or more of original body weight necessitates euthanasia; or physical condition: extreme weakness, moribund state (inability to eat/drink), or severe, untreatable infection) were applied to allow early euthanasia in accordance with IACUC guidelines. Mice were euthanized for sample collection at 52 h postinfection. In the recurrent CDI model, mice were observed for 2 weeks postinfection before euthanasia,^49^ following the same procedures as in the primary CDI model treated with G3-PLL_9_ enemas. Each group consisted of five mice, and all experiments were performed in replicate.

#### 2.3.2 Gut Microbiome Analysis and Fecal *C. difficile* Toxin Quantification

Total DNA from murine fecal samples was isolated using the High Pure PCR Template Preparation Kit (Roche) following manufacturer’s protocol. For microbiome profiling, the V3–V4 hypervariable region of the 16S rRNA gene was sequenced on an Illumina MiSeq platform with 2 × 300 nt paired-end reads. Sequence data were processed using established bioinformatic workflows,^43^ with modifications detailed in the Supporting Information. Briefly, quality-filtered reads were clustered into operational taxonomic units (OTUs) at 97% similarity against the Greengenes database (v13.8). Microbial diversity was assessed using Shannon’s diversity index (α-diversity) and Bray–Curtis dissimilarity (β-diversity).

To quantify toxigenic *C. difficile*, quantitative real-time PCR targeting the *tcdB* gene was performed on fecal DNA extracts as a molecular proxy for toxigenic strain abundance. Universal 16S rRNA bacterial primers served as an internal normalization control, and *tcdB* levels were expressed relative to total bacterial load using the 2^–ΔΔct^ method. This DNA-based approach measures the genomic burden of toxigenic strains rather than active toxin expression.

### 2.4 Statistical Analysis

The data was presented as the median ± interquartile range. Statistical analyses were conducted using GraphPad Prism version 10. The Mann–Whitney test was used for comparisons between two independent groups. For comparisons among multiple treatment groups, the Kruskal–Wallis test was followed by Dunn’s multiple comparison test. When evaluating two parameters across multiple groups, a mixed-effects model was applied, followed by Dunnett’s multiple comparison test relative to the untreated control. A *P*-value below 0.05 was considered statistically significant.

## 3 Results

### 3.1 Polypeptide Synthesis and Characterization

Figure 1 illustrates the chemical structures and synthesis of star-shaped polypeptides. The ^1^H and ^13^C NMR spectra of the initiators (PGD-G2 and PGD-G3) and star-shaped PZLL polypeptides showed that the crossover points corresponding to the ^1^H and ^13^C peaks of primary alcohol (−CH_2_OH) and secondary alcohol (−CHOH) disappeared after polymerization, suggesting successful initiation of polymerization via all hydroxyl groups (Figure S2). GPC-LS was used to determine the number-average molecular weight (*M*_*n*_) and molecular weight distribution (*M*_w_/*M*_n_) of G2-PZLL and G3-PZLL, with results summarized in Table S1. The DPs were calculated to be 10 and 9 per arm, respectively, consistent with the feed molar ratios. ^1^H NMR analysis confirmed these values, with calculated DPs aligning between polyol initiator protons and PZLL phenyl group protons (−C_6_ *H*_5_) (Table S1, Figure S3 and S4). After deprotection, ^1^H NMR showed residual Z groups below 3.0%, indicating successful deprotection (Figure S3B and S4B). The synthesized star-shaped polypeptides were designated as G2-PLL_10_ and G3-PLL_9_, respectively.

### 3.2 G3-PLL_9_ Exhibits Antimicrobial Properties Against both *C. difficile* cells and Biofilms

G3-PLL_9_ demonstrates antimicrobial activity against *C. difficile* vegetative cells. Broth dilution assays determined MIC values of 16 μM for G2-PLL_10_ and 4 μM for G3-PLL_9_, supporting the selection of G3-PLL_9_ (Figure 2A) for further investigation. Subsequent serial dilution and plating on CDC agar identified an MBC of 8 μM for G3-PLL_9_. To assess the potential for resistance development, serial passage experiments were conducted under G3-PLL_9_ exposure. *C. difficile* exhibited no more than a 2-fold increase in MIC over ten consecutive passages, with MIC values consistently remaining at 1–2 × the initial MIC (Figure S5). These findings suggest a low potential for resistance emergence. G3-PLL_9_ was also evaluated against a panel of clinical isolates representing various ribotypes, including strains resistant to commonly used anti-*C. difficile* antibiotics such as vancomycin and metronidazole. The observed MBC values for these strains ranged from 8 to 16 μM (Table S2). For comparison, vancomycin exhibited MIC and MBC values of 1 μM (1.5 μg mL^–1^) and 1.5 μM (2.2 μg mL^–1^), respectively, and fidaxomicin showed MIC and MBC values of ≤0.12 μM (≤0.125 μg mL^–1^).

**Figure 2:**
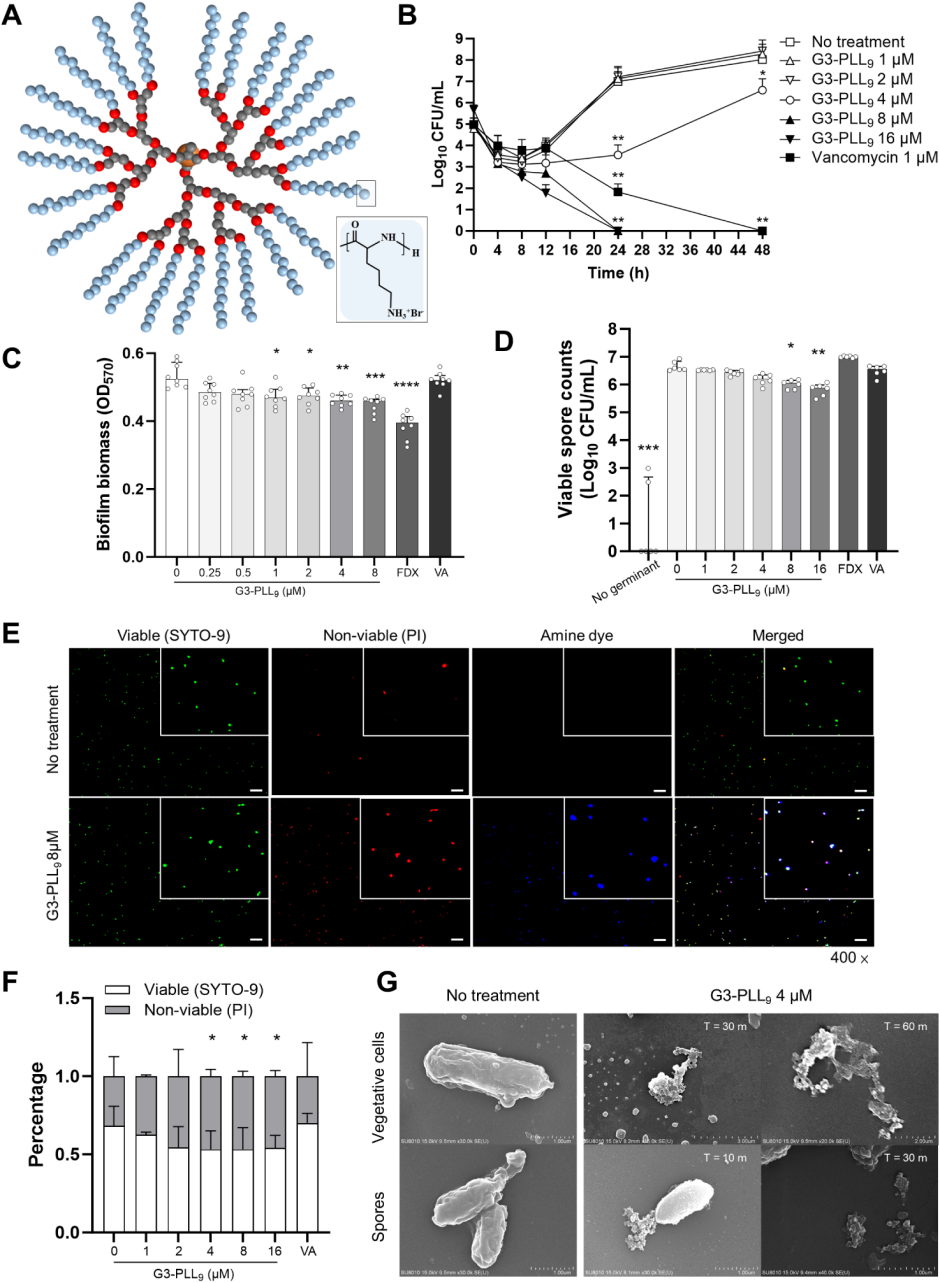
Schematic of star-shaped polypeptides G3-PLL_9_ and *in vitro* antimicrobial activity against *Clostridioides difficile*. (A) G3-PLL_9_ structure showing a dendrimer initiator (orange), carbon (black), oxygen (red), and branching poly­(l-lysine) (PLL, blue; detailed chemical structure shown in the inset). (B) Time-kill curve of *C. difficile* treated with G3-PLL_9_ at varying concentrations. Statistical significance was evaluated by comparing data to the no-treatment group at each time point using the Mann–Whitney test (*n* = 2; three independent experiments). (C) Dose-dependent disruption of *C. difficile* biofilm by G3-PLL_9_. Biofilm biomass was quantified by crystal violet staining and measured as absorbance at OD_570_. Statistical significance was assessed by comparison with the no-treatment group using the Kruskal–Wallis test followed by Dunn’s multiple-comparison test (*n* = 2; three independent experiments). (D) Viable *C. difficile* counts after G3-PLL_9_ treatment of spores and subsequent germination induced by taurocholic acid. Statistical significance was evaluated by comparison with the untreated group (second row) using the Kruskal–Wallis test followed by Dunn’s multiple-comparison test (*n* = 2; three independent experiments). (E) Fluorescence microscopy of *C. difficile* spores treated with G3-PLL_9_. Green, viable spores (SYTO-9); red, nonviable spores (PI); blue, G3-PLL_9_ (Alexa Fluor 350). White spots indicate nonviable spores interacting with G3-PLL_9_. Scale bars, 20 μm. (F) Quantification of fluorescence microscopy images showing the percentage of viable and nonviable spores relative to total spore count. Statistical significance was evaluated by comparing nonviable cells of each treatment group to the untreated group using the Mann–Whitney test (*n* = 10 per condition). (G) Scanning electron microscopy images of *C. difficile* cells and spores, untreated (left) and treated with G3-PLL_9_ for indicated durations. Early roughening of the spore outer layer (10 min) and size reduction (30–60 min) are observed. Data in (B–D and F) are presented as median ± interquartile range. Fidaxomicin (FDX) and vancomycin (VA) at their minimum inhibitory concentrations were included as antibiotic comparators. *P < 0.05, **P < 0.01, ***P < 0.001, ****P < 0.0001. CFU, colony-forming unit.

In time-kill analyses, G3-PLL_9_ at concentrations of 8 μM or higher markedly reduced bacterial counts at 24 h and maintained sustained inhibitory activity throughout the 48 h observation period (Figure 2B). At 4 μM or lower, partial inhibition was observed within the first 24 h; however, *C. difficile* recovered beyond this period (Figure 2B). Notably, at higher inoculum levels (10^6^–10^7^ CFU mL^–1^), G3-PLL_9_ at 8 μM or higher maintained its inhibitory effects throughout 48 h, contrasting with vancomycin at its MIC (1 μM) (Figure S6).

Additionally, G3-PLL_9_ significantly disrupted *C. difficile* biofilms in a dose-dependent manner at concentrations ≥1 μM. While fidaxomicin exhibited comparable antibiofilm activity, vancomycin showed no significant effect under the same conditions (Figure 2C). Importantly, G3-PLL_9_ dismantled biofilm at sub-MIC concentrations as low as 1 μM. These findings suggest that G3-PLL_9_ exhibits inhibitory effects on *C. difficile* vegetative cells at concentrations of 4 μM or higher, and on biofilms at concentrations of 1 μM or higher, highlighting its efficacy against both *C. difficile* vegetative cells and biofilms.

To investigate the antimicrobial mechanism of G3-PLL_9_ against *C. difficile*, RNA sequencing analysis was performed. Differential gene expression analysis identified significant changes in genes encoding sensor histidine kinases and response regulator transcription factors (Table S3) in *C. difficile* following G3-PLL_9_ treatment. KEGG pathway analysis highlighted enrichment in sulfur metabolism and cysteine/methionine metabolism pathways (Table S4, Figure S7A), while GO analysis showed significant enrichment in transcriptional regulation and DNA binding processes (Table S4, Figure S7B). These exploratory results suggest G3-PLL_9_ treatment is associated with transcriptomic changes in genes involved in metabolic and regulatory pathways in *C. difficile*, while direct mechanistic effects were not assessed in this study.

### 3.3 G3-PLL_9_ Inhibits *C. difficile* Spores through Structure Destruction

The inhibitory effects of G3-PLL_9_ on *C. difficile* spores were evaluated using multiple complementary methods. At concentrations of 4 μM or higher, G3-PLL_9_ induced approximately a one-log reduction in viable bacterial colonies following treatment of spores (Figure 2D). Fluorescence microscopy revealed an increased number of nonviable spores, indicated by red fluorescence, after G3-PLL_9_ treatment (Figure 2E, second column). The polypeptide, conjugated with an amine-reactive blue dye, appeared as white spots in the merged images, suggesting close association with spore surfaces (Figure 2E, third and fourth column). Quantitative analysis of fluorescence images further confirmed a significant increase in nonviable spores at concentrations of 4 μM or higher (Figure 2F). In contrast, vancomycin (1 μM) showed no observable effect on spore viability (Figure 2D and 2F).

SEM images revealed that G3-PLL_9_ caused surface roughening of spores within 10 min of treatment at 4 μM, followed by disintegration and shrinkage of both spores and vegetative cells (Figure 2G). G3-PLL_9_ induced distinct morphological alterations that were clearly distinguishable from those elicited by vancomycin or fidaxomicin (Figure S8). Consistently, ATP-based viability assays (BacTiter-Glo) showed a dose-dependent reduction in viable spore counts following G3-PLL_9_ treatment, with the most pronounced effect observed at 16 μM (Figure S9), corroborating the structural damage observed by SEM. Notably, G3-PLL_9_ did not inhibit spore germination (Figure S10), indicating that its spore-killing mechanism involves structural damage rather than germination inhibition.

### 3.4 G3-PLL_9_ Demonstrates Therapeutic Efficacy and a Favorable Safety Profile *In Vivo*

The *in vitro* antimicrobial activity of G3-PLL_9_ against both vegetative cells and spores of *C. difficile* was established in earlier experiments. Following confirmation that G3-PLL_9_ did not induce increased hemolysis at therapeutic concentrations (Figure S11), the *in vivo* efficacy was evaluated using a mouse model of CDI (Figure 3A). Oral administration of G3-PLL_9_ showed limited effectiveness compared to enema delivery (Figure S12), likely due to degradation by gastric acid. Consequently, subsequent experiments were conducted using anal delivery. Compared to untreated controls, G3-PLL_9_ (1 μM) therapy attenuated weight loss (−6.7% vs −14.0%, *P* < 0.001; Figure 3B) and improved symptom severity, colon morphology (Figure 3C), colon length (8.2 cm vs 6.1 cm, *P* = 0.05; Figure 3D), and cecum weight (1.6 g vs 1.0 g, *P* < 0.0001; Figure 3E) among mice with CDI. Histological analysis revealed reduced mucosal damage and neutrophil infiltration in the G3-PLL_9_ 1 μM group (histology injury score 4.2 vs 5.8, *P* = 0.02; Figure 3F–I). In addition, serum biochemistry analysis indicated lower aspartate aminotransferase levels in the G3-PLL_9_ 1 μM group compared with untreated mice (Figure 3L). Additionally, both G3-PLL_9_ and vancomycin were associated with lower fecal *tcdB* DNA levels, a surrogate marker of *C. difficile* bacterial burden, compared with untreated controls, although these differences did not reach statistical significance (Figure S13).

**Figure 3:**
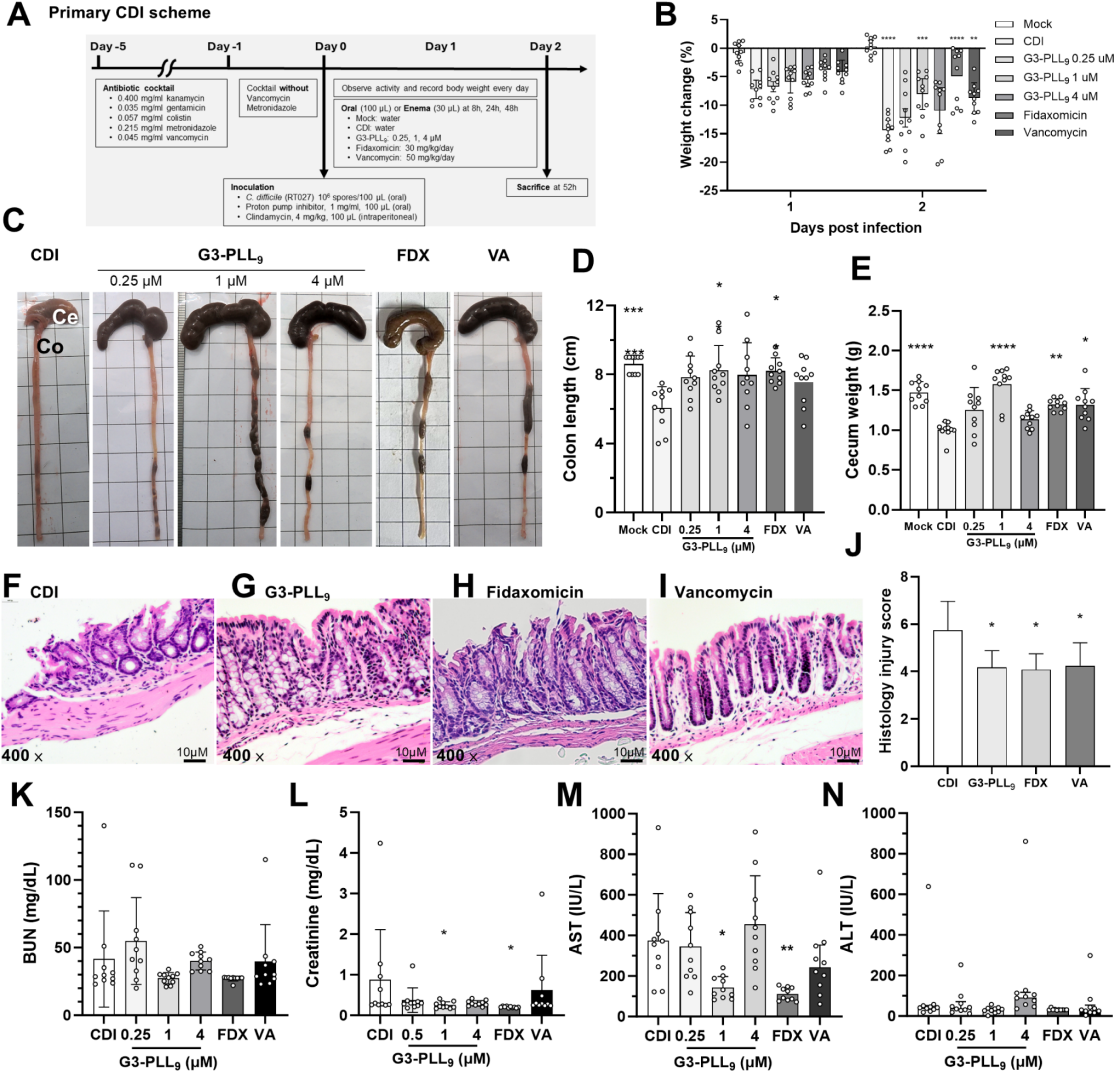
*In vivo* therapeutic efficacy of G3-PLL_9_ in a mouse model of *Clostridioides difficile* infection (CDI). (A) Schematic of the primary CDI mouse model protocol. (B) Percentage change in body weight relative to the day of infection. Statistical analysis was performed using a mixed-effects model followed by Dunnett’s multiple comparison test relative to the untreated CDI group (*n* = 5; two independent experiments). (C) Representative gross morphology of mouse cecum (Ce) and colon (Co) in each group. FDX, fidaxomicin; VA, vancomycin. (D–E) Therapeutic response indicators: colon length (D) and cecum weight (E) (*n* = 5; two independent experiments). (F–I) Histopathological analysis of colonic sections from CDI-infected mice: no treatment (F), G3-PLL_9_ (G), fidaxomicin (H), and vancomycin (I). Scale bars, 100 μm. (J) Histological injury scores based on tissue damage, mucosal edema, and neutrophil infiltration (six high-power fields per sample). (K–N) Safety assessment via serum biochemistry: blood urea nitrogen (BUN) (K), creatinine (L), aspartate aminotransferase (AST) (M), and alanine transaminase (ALT) (N) (*n* = 5; two independent experiments). Data in (B, D–E, J–N) are presented as median ± interquartile range. Statistical analyses for (D–E, J–N) were performed using the Kruskal–Wallis test with Dunn’s multiple comparison test relative to the untreated CDI group. *P < 0.05, **P < 0.01, ***P < 0.001, ****P < 0.0001.

In a recurrent CDI mouse model (Figure 4A), G3-PLL_9_ (−9.0%, *P* < 0.01), fidaxomicin (−4.7%, *P* < 0.001), and vancomycin (−4.1%, *P* < 0.001) groups all demonstrated less weight loss than untreated controls (−16.3%) during the acute phase within the first 2 days of infection (Figure 4B). Recurrence was observed approximately 1 week postinfection, characterized by diarrhea and additional weight loss. During this phase, the vancomycin group exhibited a 60% death rate, −14.3% weight loss, and the lowest cecum weight (0.42 g). In contrast, both G3-PLL_9_ and fidaxomicin groups showed a lower death rate of 40%, attenuated weight loss (Figure 4B), and preserved cecum weight (0.57 and 0.63 g; Figure 4F). Although the vancomycin group had lower toxin levels at the end of treatment, a rebound increase in toxin load was observed by the end of the observation period (Figure 4D). These findings suggest that G3-PLL_9_ treatment was associated with reduced recurrence-related symptoms, potentially mediated by its effects on *C. difficile* spores; however, further investigation is warranted to elucidate the underlying mechanisms.

**Figure 4:**
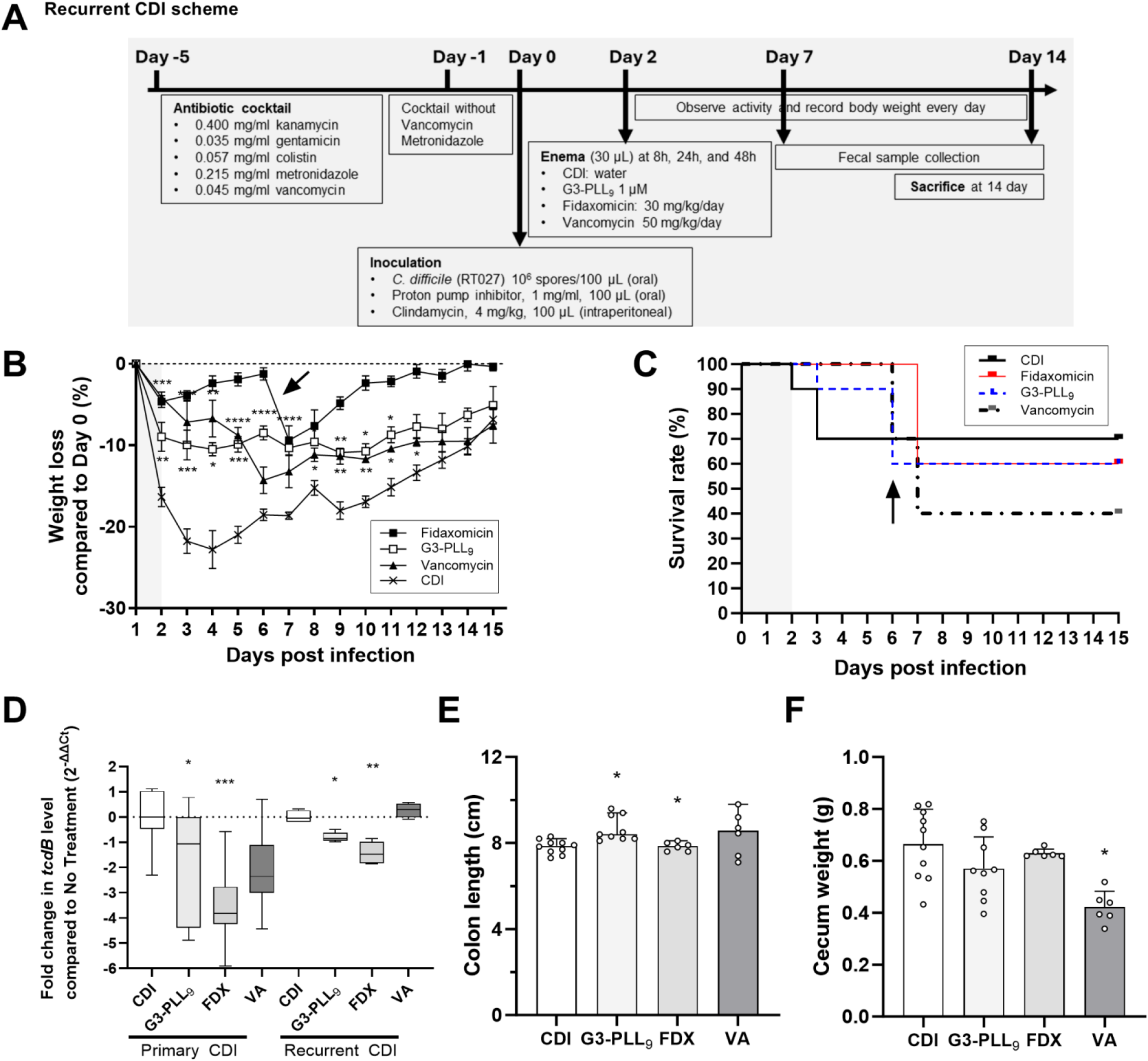
*In vivo* therapeutic effects of G3-PLL_9_ in a mouse model of recurrent *Clostridioides difficile* infection (CDI). (A) Schematic of the recurrent CDI mouse model protocol. (B–C) Percentage change in body weight (B) and survival curve (C) over 15 days relative to the day of infection. Shaded areas indicate treatment periods; the arrow marks symptom recurrence. Statistical analysis in (B) was performed using a mixed-effects model with Dunnett’s multiple comparison test relative to the untreated CDI group (*n* = 5; two independent experiments). (D) Stool *tcdB* levels quantified by real-time PCR during primary and recurrent CDI. For each infection stage, treatment groups were compared to the corresponding untreated CDI group using the Mann–Whitney test (*n* = 3; two independent experiments). (E–F) Therapeutic response indicators: colon length (E) and cecum weight (F) (*n* = 5; two independent experiments). Data in (B, D–F) are presented as median ± interquartile range. Statistical analyses for (E–F) were performed using the Kruskal–Wallis test with Dunn’s multiple comparison test relative to the untreated CDI group. *P < 0.05, **P < 0.01, ***P < 0.001.

### 3.5 Oral Delivery Enabled by Cysteamine-Modified Hyaluronic Acid/G3-PLL_9_ Microgel

To enhance the potential clinical applicability of G3-PLL_9_, the Cys-modified HA was used to complex with G3-PLL_9_ to form microgels via electrostatic interactions as schematic illustrated in Figure 5A. It is worth noting that the mixing of unmodified HA and G3-PLL_9_ would result in precipitation. The conjugation of Cys onto HA would lower the charge density on HA, which could afford the formation of colloidally stable HA/G3-PLL_9_ microgels. Based on the ^1^H NMR spectrum (Figure S14), the DS of Cys was determined to be 39.9% using the method described in a previous study.^50^ For the resulting HA/G3-PLL_9_ microgel solution, the concentration of G3-PLL_9_ was 0.45 mg mL^–1^. Based on the DLS analysis, the particle size, PDI, and zeta potential of the microgels were measured to be 355.4 ± 40.4 nm, 0.354 ± 0.001 and 39.6 ± 1.4 mV, respectively (Figure S15A). The HA/G3-PLL_9_ microgels remained stably suspended in aqueous solution (pH 7.4) at room temperature for at least 2 weeks. Notably, their particle size remained stable for up to 5 days even under acidic (pH 5.1) or basic (pH 8.4) conditions, after which gradual degradation of the microgels was observed (Figure S15B–D).

**Figure 5:**
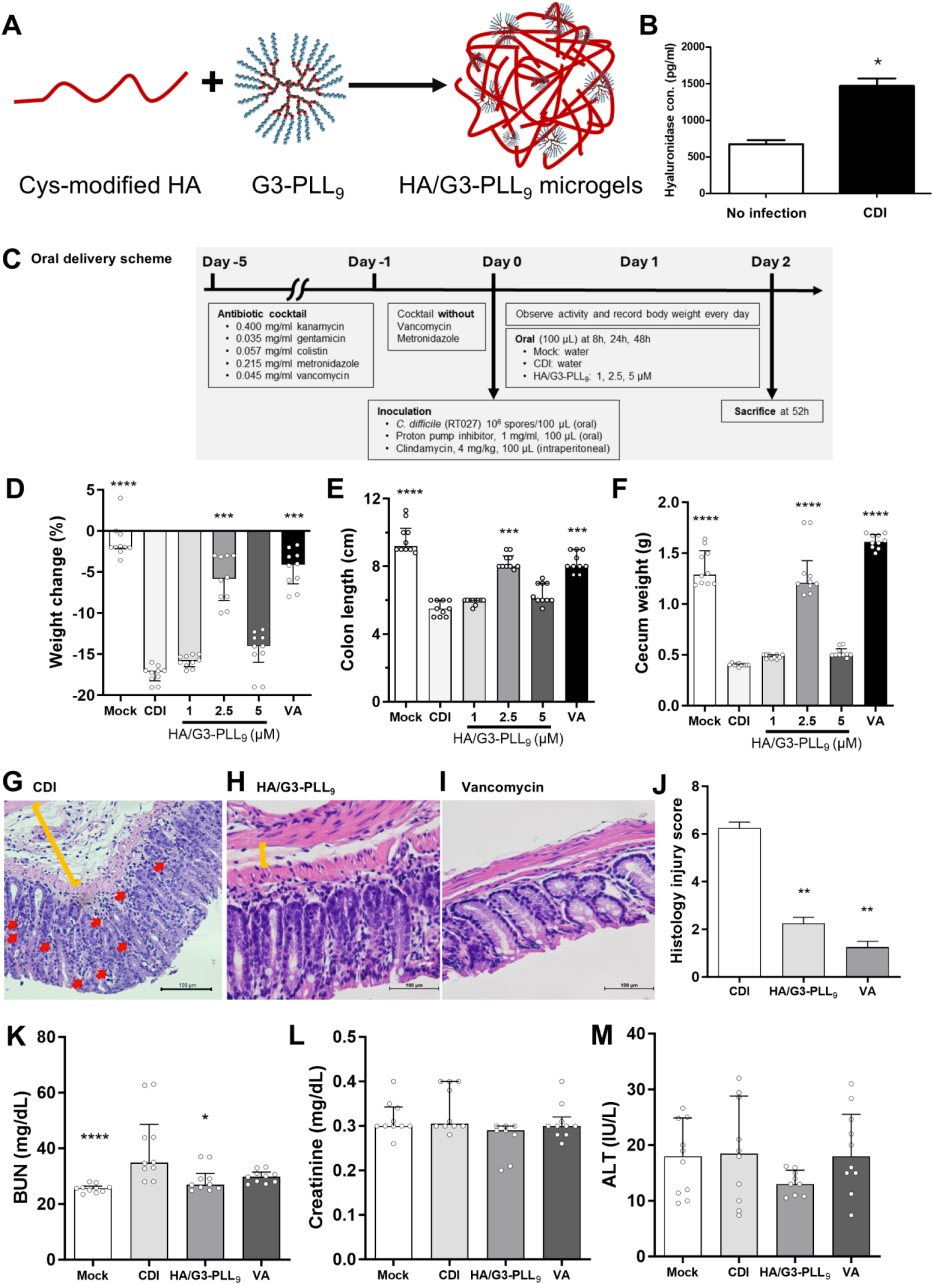
*In vivo* therapeutic efficacy of orally administered hyaluronic acid (HA)/G3-PLL_9_ microgels in a mouse model of *Clostridioides difficile* infection (CDI). (A) Schematic of HA/G3-PLL_9_ microgel synthesis via electrostatic interaction. Cys, cysteamine. (B) Colonic hyaluronidase levels in uninfected and CDI-infected mice (*n* = 3; two independent experiments). (C) Schematic of the oral delivery protocol for HA/G3-PLL_9_ microgels. (D–F) Therapeutic efficacy indicators: body weight change relative to the day of infection (D), colon length (E), and cecum weight (F) (*n* = 5; two independent experiments). (G–I) Representative histopathological images of colonic sections from CDI-infected mice receiving no treatment (G), HA/G3-PLL_9_ microgels (H), or vancomycin (I). Scale bars, 100 μm. (J) Histological injury scores based on tissue damage, mucosal edema, and neutrophil infiltration (six high-power fields per sample). (K–M) Safety assessment of HA/G3-PLL_9_ microgels: serum blood urea nitrogen (BUN) (K), creatinine (L), and alanine transaminase (ALT) (M) levels (*n* = 5; two independent experiments). Data in (B, D–F, and J–M) are presented as median ± interquartile range. Statistical comparisons between treatment groups and the CDI group were performed using the Kruskal–Wallis test with Dunn’s post hoc test. *P < 0.05, **P < 0.01, ***P < 0.001, ****P < 0.0001.

In the CDI mouse model, increased hyaluronidase production was observed in the colon compared to uninfected mice (Figure 5B). This localized enzyme activity may facilitate the degradation of HA/G3-PLL_9_ microgels at the infection site (Figure S15E), minimizing off-target effects. HA/G3-PLL_9_ microgels were then administered orally following the established protocol for the CDI mouse model (Figure 5C). At a concentration of 2.5 μM, oral administration of HA/G3-PLL_9_ microgels demonstrated therapeutic effects, preserving body weight (−5.8% vs −17%, *P* < 0.01; Figure 5D), colon length (8.0 cm vs 5.5 cm, *P* < 0.001; Figure 5E), cecum weight (1.2 g vs 0.4 g, *P* < 0.0001; Figure 5F), and, and reducing tissue damage (histology injury score 2.3 vs 6.3, *P* < 0.01; Figure 5G–J) compared to untreated control. Furthermore, safety analyses revealed no increased liver or kidney injury compared to other treatment groups (Figure 5K–M). The results indicate that oral administration of HA/G3-PLL_9_ microgels produce similar treatment effects to G3-PLL_9_ enema.

### 3.6 G3-PLL_9_ Preserves Gut Microbiota Compared to Vancomycin in CDI Treatment

Preliminary tests indicated that G3-PLL_9_ exhibits limited antimicrobial activity against representative gut commensals, including *Escherichia coli*, *Klebsiella pneumoniae*, and *Staphylococcus aureus* (Table S2). Analysis of fecal microbiota revealed treatment-associated differences in taxonomic composition among groups (Figure 6A). Vancomycin-treated samples exhibited enrichment of potentially pathogenic genera (*Escherichia*, *Enterococcus*, *Proteus*) while depleting beneficial genera (*Bacteroides*, *Parabacteroides*); in contrast, G3-PLL_9_ and HA/G3-PLL_9_ groups showed enrichment of commensal genera including *Parabacteroides* and *Bacteroides* (Figure 6A, Figure S16). Alpha diversity analysis using the Shannon index revealed that G3-PLL_9_ and HA/G3-PLL_9_ treatments maintained microbial diversity comparable to fidaxomicin (G3-PLL_9_ vs fidaxomicin, *P* = 0.748; HA/G3-PLL_9_ vs fidaxomicin, *P* = 0.409) and significantly higher than vancomycin (G3-PLL_9_ vs vancomycin, *P* = 0.009; HA/G3-PLL_9_ vs vancomycin, *P* = 0.029; Figure 6B). Beta diversity analysis showed that vancomycin-treated samples formed a distinct cluster, whereas G3-PLL_9_, HA/G3-PLL_9_, and fidaxomicin groups clustered closer to the Mock and CDI groups (PERMANOVA: G3-PLL_9_ vs vancomycin, *P* = 0.007; HA/G3-PLL_9_ vs vancomycin, *P* = 0.007; Figure 6C), with no significant difference between G3-PLL_9_ and HA/G3-PLL_9_ groups (*P* = 0.603), indicating comparable microbiota-sparing effects between enema and oral delivery. Collectively, these data suggest that under the conditions tested, G3-PLL_9_-based treatments are associated with less microbiota disruption than vancomycin, with efficacy comparable to fidaxomicin.

**Figure 6:**
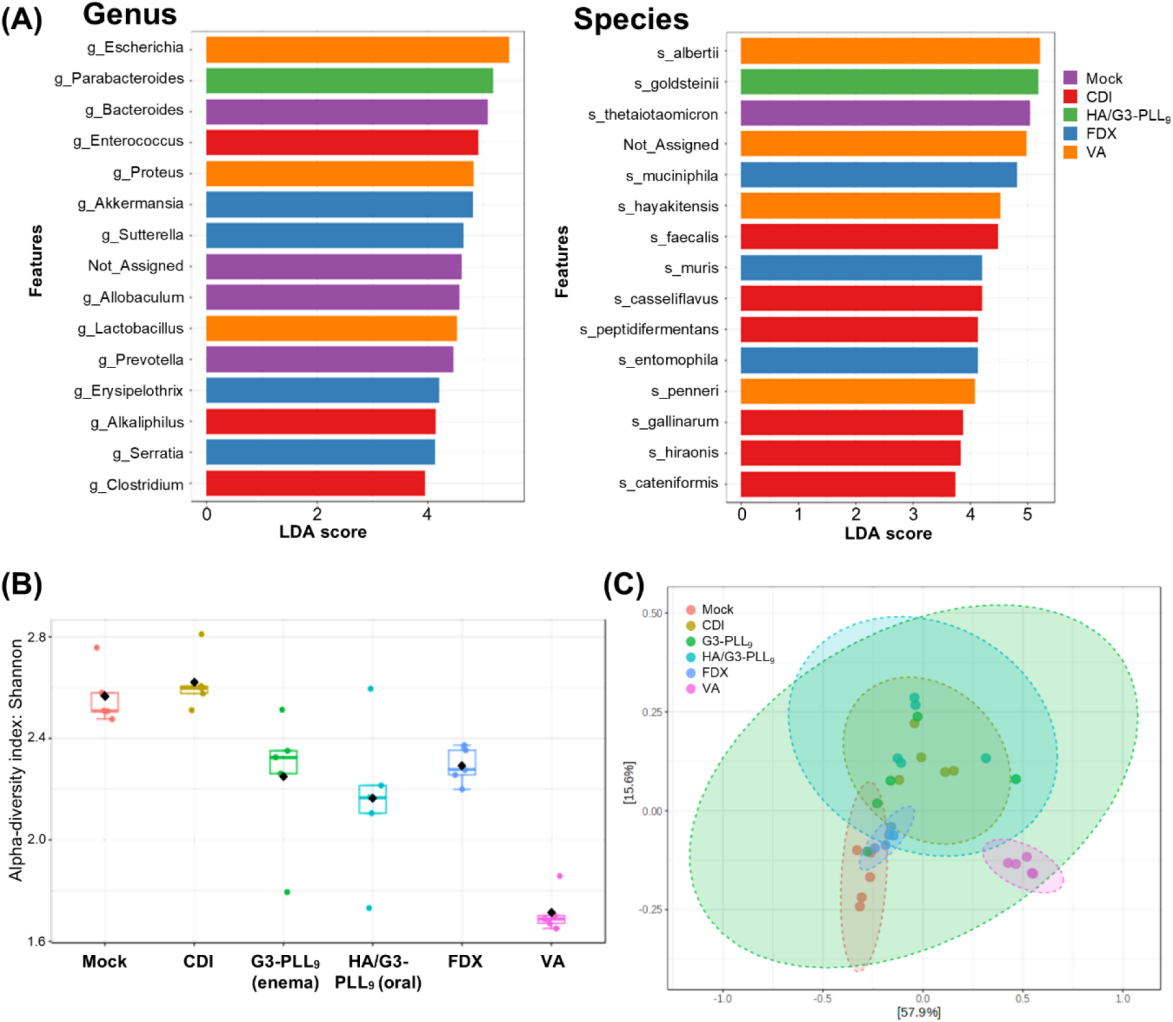
Microbiota composition and diversity analysis after treatment for *Clostridioides difficile* infection (CDI). Fecal specimens were collected at the time of sacrifice. (A) Linear discriminant analysis Effect Size (LEfSe) identifying differentially abundant taxa at the genus (left) and species (right) levels (LDA score >2.0, *P* < 0.05). Colors indicate the treatment group in which each taxon was enriched. (B) Alpha diversity analysis using the Shannon index. G3-PLL_9_ and HA/G3-PLL_9_ treatments preserved microbial diversity comparable to fidaxomicin (FDX) (G3-PLL_9_ vs FDX, *P* = 0.748; HA/G3-PLL_9_ vs FDX, *P* = 0.409) and significantly higher than vancomycin (VA) (G3-PLL_9_ vs VA, *P* = 0.009; HA/G3-PLL_9_ vs VA, *P* = 0.029; Kruskal–Wallis test). (C) Principal coordinates analysis (PCoA) based on Bray–Curtis dissimilarity illustrating beta diversity. Each point represents an individual sample, and ellipses indicate 95% confidence intervals. G3-PLL_9_ and HA/G3-PLL_9_ groups clustered distinctly from vancomycin (VA) (PERMANOVA: G3-PLL9 vs VA, *P* = 0.007; HA/G3-PLL_9_ vs VA, *P* = 0.007).

## 4 Discussion

Current treatment options for *C. difficile* infection include vancomycin, metronidazole, and fidaxomicin.^10^ In this study, vancomycin and fidaxomicin were selected as comparators because of its long-standing clinical use and well-characterized activity against vegetative *C. difficile*. Vancomycin and fidaxomicin inhibit bacterial cell wall synthesis and is therefore effective primarily against actively dividing cells. In contrast, star-shaped polypeptides are designed to mimic natural antimicrobial polymers and are proposed to act independently of bacterial growth cycle. This study evaluated the antimicrobial properties of the star-shaped polypeptide G3-PLL_9_ against *C. difficile*. To minimize bias associated with visual MIC determination due to the intrinsic opacity of polypeptides, both MIC and MBC values were reported. The MIC of G3-PLL_9_ was determined to be 4 μM, while the MBC, confirmed by additional colony enumeration, was 8 μM. Compared with vancomycin (MIC 1 μM; 1.45 μg mL^–1^), G3-PLL_9_ at 4–8 μM exhibited bactericidal and sporicidal activity *in vitro* and disrupted *C. difficile* biofilms at sub-MIC concentrations. Consistent with previous reports, fidaxomicin exhibited potent antimicrobial activity against *C. difficile*;^51^ notably, our data further demonstrated its efficacy in disrupting established biofilms.^51^ However, neither fidaxomicin nor vancomycin effectively eradicated spores. In CDI mouse models, G3-PLL_9_ alleviated symptoms, reduced weight loss, minimized tissue damage, mitigated recurrence symptoms, and exhibited an acceptable safety profile. Additionally, its complexation with HA to form HA/G3-PLL_9_ microgels facilitated oral administration and enhanced targeted drug delivery, reducing off-target effects and improving therapeutic potential. Compared with vancomycin, G3-PLL_9_ treatment was associated with less pronounced alterations in gut microbiome composition, consistent with vancomycin’s known contribution to dysbiosis and recurrence risk.^15,16^ Collectively, these findings support star-shaped polypeptides as a candidate antimicrobial platform with potential advantages in both efficacy and microbiome preservation.

The underlying mechanisms proposed herein require further validation through broader experimental models to ensure a comprehensive interpretation of G3-PLL_9_ activity. SEM analysis revealed extensive morphological alterations in both *C. difficile* vegetative cells and spores following G3-PLL_9_ treatment, which is consistent with the membrane-disruptive behavior reported for cationic antimicrobial polymers.^17–19,24,25^ However, these observations do not directly establish a specific molecular mechanism. Rather, our mechanistic interpretations are informed by prior studies on structurally nanoengineered antimicrobial polypeptide polymers (SNAPPs), in which membrane disruption has been demonstrated using detailed biophysical and ultrastructural analyses.^17,19^ Similar electron microscopy phenotypes have been reported for SNAPPs against other bacterial pathogens, supporting the plausibility of a related mode of action.^19,24^ While previous work has shown that increased architectural complexity, such as higher arm number, can enhance antimicrobial potency through multivalent electrostatic interactions,^18,19,22^ these principles have not been directly validated for *C. difficile* in the present study. Likewise, the proposed interaction between G3-PLL_9_ and negatively charged components of the spore exosporium, such as BclA, remains speculative and is inferred from established spore surface structure.^52,53^ Future investigations incorporating biophysical measurements will be required to conclusively define the antimicrobial and sporicidal mechanisms of G3-PLL_9_.

The transcriptomic data provided an exploratory overview of the cellular response, intended to facilitate hypothesis-driven investigations into the specific antimicrobial pathways targeted by G3-PLL_9_. Differential gene expression analysis suggested that G3-PLL_9_ exposure may be associated with alterations in metabolic and regulatory pathways in *C. difficile*, including those related to cysteine metabolism, histidine kinase-mediated signaling, and oxidative stress responses.^54,55^ Histidine kinase signaling is therefore proposed as a potential pathway of interest, supported by prior reports describing antimicrobial peptides that target the histidine kinase YycG in *Staphylococcus aureus*.^56^ While G3-PLL_9_ appears to go beyond agents that primarily inhibit spore germination or target antivirulence pathways,^57–62^ its ability to eradicate bacterial cells, spores, and biofilms, along with its potential modulation of metabolic pathways, could play a role in reducing CDI persistence and recurrence.

The complexation of HA protected G3-PLL_9_ from premature degradation, allowing it to reach the colon intact.^30,34^ This delivery system exploited elevated hyaluronidase levels present in inflamed colonic tissue, facilitating site-specific release and enhancing therapeutic efficacy via oral administration.^31^ To account for the prolonged transit time through the gastrointestinal tract and potential electrostatic interactions between HA and G3-PLL_9_, higher doses of HA/G3-PLL_9_ were used for oral delivery (1, 2.5, and 5 μM) compared to direct enema administration (0.25, 1, and 4 μM). In the CDI mouse model, HA/G3-PLL_9_ microgels demonstrated therapeutic efficacy at 2.5 μM when administered orally. However, at 1 μM, the orally delivered HA/G3-PLL_9_ microgels were less effective than G3-PLL_9_ delivered via enema. This reduced efficacy is likely attributable to factors such as extended gastrointestinal transit time, dilution effects, and altered charge profiles of G3-PLL_9_ during oral delivery. At 5 μM, reduced efficacy was observed, possibly due to self-aggregation and precipitation, which impaired the bioavailability.

Regarding safety, the *in vitro* hemolysis assay demonstrated no increased hemolysis at the therapeutic doses used in this study. In CDI mouse models, G3-PLL_9_ did not appear to cause renal or liver damages. Histological analysis of colon tissues also revealed no additional damage compared to vancomycin treatment. These findings are consistent with previous studies on other SNAPPs in mouse models.^18^ However, further research is warranted to comprehensively evaluate the safety profile of G3-PLL_9_ beyond renal and liver functions.

This study has several limitations. First, antimicrobial susceptibility testing was not performed using the CLSI-recommended agar dilution method for anaerobic bacteria, because G3-PLL_9_ is a peptide-based polymer, preliminary experiments indicated that agar preparation involving autoclaving could adversely affect its structural integrity and antimicrobial activity. Therefore, a broth dilution assay, which is also commonly used for antimicrobial susceptibility testing,^43^ was employed to ensure a reliable assessment of antimicrobial effects. Second, while histidine kinase signaling and oxidative stress response were proposed as potential contributors to the bactericidal activity of G3-PLL_9_, the specific molecular pathways involved were not directly investigated. Third, the mechanisms underlying the observed sporicidal and antibiofilm activities were not elucidated in this study. Fourth, the localization of G3-PLL_9_ release from HA/G3-PLL_9_ microgels and the effects of gastrointestinal enzymes (e.g., pepsin and trypsin) were not directly assessed. Nevertheless, *in vitro* characterization demonstrated that the microgels are highly sensitive to hyaluronidase-mediated degradation, enabling the efficient liberation of G3-PLL_9_ within 1 h—a time frame that aligns with the requirements for targeted intervention in the lower gastrointestinal tract. Furthermore, the particle size of HA/G3-PLL_9_ microgels remained stable for 5 days under both acidic and basic conditions, suggesting that the formulation can withstand gastrointestinal transit. Consistently, therapeutic outcomes in the mouse CDI model following oral administration of HA/G3-PLL_9_ microgels were comparable to those achieved with enema delivery, supporting the feasibility and effectiveness of this oral delivery strategy. Finally, as this study represents a proof-of-concept investigation, long-term safety, effects on individual commensal gut taxa, and genomic analyses of potential resistance-related mutations in *C. difficile* have not been explored. However, serial passage experiments indicated a low propensity for resistance development under the conditions tested.

This study highlights the potential of the cationic, star-shaped polypeptide G3-PLL_9_ as a novel therapeutic strategy for CDI. G3-PLL_9_ demonstrated potent antimicrobial activity by effectively targeting *C. difficile* vegetative cells, spores, and biofilms. Unlike conventional antibiotics such as vancomycin, which inhibit cell wall synthesis, G3-PLL_9_ disrupts bacterial morphology through multifaceted interactions with cell membranes and metabolic pathways, potentially reducing the risk of antimicrobial resistance.

In CDI mouse models, G3-PLL_9_ alleviated symptoms and showed therapeutic efficacy comparable to vancomycin. Additionally, its complexation with HA enabled oral administration, enhanced targeted drug delivery to inflamed infection sites, and minimized off-target effects. Notably, G3-PLL_9_ caused less disruption to the intestinal microbiota and had minimal impact on organ function compared to vancomycin.

These findings underscore the promise of G3-PLL_9_ as an innovative antimicrobial agent with a multifaceted mechanism of action, improved microbiome preservation, and potential for oral administration, offering a compelling alternative to current CDI treatments.

## 5 Conclusions

This study demonstrates that the cationic, star-shaped polypeptide G3-PLL_9_ represents a promising antimicrobial platform for the treatment of CDI. G3-PLL_9_ exhibited potent *in vitro* activity against *C. difficile* vegetative cells, spores, and biofilms, three key factors underlying persistence and recurrence of CDI. Notably, its bactericidal and sporicidal effects, together with its ability to disrupt established biofilms at sub-MIC concentrations, distinguish it from conventional antibiotics such as vancomycin and fidaxomicin, which primarily target actively dividing cells and have limited activity against spores and biofilms. *In vivo*, G3-PLL_9_ treatment improved clinical outcomes in both primary and recurrent CDI mouse models, including attenuation of weight loss, preservation of intestinal morphology, and reduction of histological damage. Importantly, G3-PLL_9_ was associated with reduced recurrence-related disease severity and demonstrated a favorable safety profile without evident hepatotoxicity or nephrotoxicity. In addition, microbiome analyses indicated that G3-PLL_9_ preserved gut microbial diversity to a greater extent than vancomycin, supporting its potential advantage in minimizing dysbiosis-associated recurrence.

To enhance clinical applicability, complexation with cysteamine-modified HA enabled the formation of HA/G3-PLL_9_ microgels for oral delivery. This formulation demonstrated stability under gastrointestinal conditions and facilitated inflammation-responsive release in the colon, achieving therapeutic efficacy comparable to direct enema administration while minimizing off-target effects. Collectively, these findings establish G3-PLL_9_ as a multifunctional antimicrobial agent capable of targeting multiple pathogenic states of *C. difficile*, while offering advantages in microbiome preservation and drug delivery. This work provides a proof-of-concept for the development of star-shaped polypeptide-based therapeutics and supports further investigation into their clinical translation for CDI and other difficult-to-treat infections.

## Supplementary Material





## Data Availability

The data that support the findings of this study are available from the corresponding author upon reasonable request.

## Notes

Materials and methods; GPC analysis and NMR spectra of star-shaped PZLL; MBCs of G3-PLL_9_; list of 45 differentially expressed genes; enrichment analysis of differentially expressed genes; synthesis scheme of cysteamine-modified HA; ^1^H and^13^ C NMR spectra of PGD-G3, G3-PZLL_9_, G2-PZLL_10_, G2-PLL_10_, G3-PZLL_9_, G3-PLL_9_, HA, and cysteamine-modified HA; Serial passages of *CD* (strain JIK 8284) after exposure to sub-MIC concentrations of G3-PLL_9_; time-kill growth curve analysis of G3-PLL_9_ against high-inoculum *CD*; Kyoto Encyclopedia of Genes and Genomes (KEGG) and Gene Ontology (GO) enrichment analysis; SEM images of *CD* vegetative cells and spores; ATP-based viability assay; Germination inhibition assay of G3-PLL_9_ against *CD* spores; hemolytic activity of G3-PLL_9_ compared to vancomycin; efficacy of G3-PLL_9_ in CDI treatment via oral versus enema administration; quantification of stool *tcdB* levels using real-time polymerase chain reaction; characterization of HA/G3-PLL_9_ microgels; microbiota composition after treatment for CDI (PDF)
Raw data file (XLS)

## References

[1] Akorful R. A. A., Odoom A., Awere-Duodu A., Donkor E. S. (2025). The Global Burden
of *Clostridioides difficile* Infections, 2016–2024:
A Systematic Review and Meta-Analysis. Infect.
Dis. Rep..

[2] Bella S. D., Sanson G., Monticelli J., Zerbato V., Principe L., Giuffrè M., Pipitone G., Luzzati R. (2024). *Clostridioides
difficile* Infection: History, Epidemiology, Risk Factors,
Prevention, Clinical Manifestations, Treatment, and Future Options. Clin. Microbiol. Rev..

[3] Borren N. Z., Ghadermarzi S., Hutfless S., Ananthakrishnan A. N. (2017). The Emergence
of *Clostridium difficile* Infection in Asia: A Systematic
Review and Meta-Analysis of Incidence and Impact. PLoS One.

[4] Kelly C. R., Allegretti J. R. (2023). Review Article: Gastroenterology
and *Clostridium
difficile* Infection: Past, Present, and Future. Clin. Infect. Dis..

[5] Maillard J. Y., Pascoe M. (2024). Disinfectants and Antiseptics: Mechanisms of Action
and Resistance. Nat. Rev. Microbiol..

[6] Coullon H., Rifflet A., Wheeler R., Janoir C., Boneca I. G., Candela T. (2018). N-Deacetylases Required for Muramic-δ-Lactam
Production Are Involved in *Clostridium difficile* Sporulation,
Germination, and Heat Resistance. J. Biol. Chem..

[7] Paredes-Sabja D., Shen A., Sorg J. A. (2014). *Clostridium difficile* Spore Biology: Sporulation, Germination, and Spore Structural Proteins. Trends Microbiol..

[8] Kordus S. L., Thomas A. K., Lacy D. B. (2022). *Clostridioides difficile* Toxins: Mechanisms of Action and
Antitoxin Therapeutics. Nat. Rev. Microbiol..

[9] Vuotto C., Donelli G., Buckley A., Chilton C. (2024). *Clostridioides
difficile* Biofilm. Adv. Exp. Med. Biol..

[10] Johnson S., Lavergne V., Skinner A. M., Gonzales-Luna A. J., Garey K. W., Kelly C. P., Wilcox M. H. (2021). Clinical
Practice
Guideline by the Infectious Diseases Society of America (IDSA) and
Society for Healthcare Epidemiology of America (SHEA): 2021 Focused
Update Guidelines on Management of *Clostridioides difficile* Infection in Adults. Clin. Infect. Dis..

[11] Budi N., Godfrey J. J., Safdar N., Shukla S. K., Rose W. E. (2021). Omadacycline
Compared to Vancomycin When Combined with Germinants to Disrupt the
Life Cycle of *Clostridioides difficile*. Antimicrob. Agents Chemother..

[12] Krutova M., Wilcox M., Kuijper E. (2022). *Clostridioides
difficile* Infection: Are the Three Currently Used Antibiotic
Treatment Options
Equal from Pharmacological and Microbiological Points of View?. Int. J. Infect Dis..

[13] James G. A., Chesnel L., Boegli L., DeLancey
Pulcini E., Fisher S., Stewart P. S. (2018). Analysis of *Clostridium difficile* Biofilms: Imaging and Antimicrobial
Treatment. J. Antimicrob. Chemother..

[14] Đapa T., Leuzzi R., Ng Y. K., Baban S. T., Adamo R., Kuehne S. A., Scarselli M., Minton N. P., Serruto D., Unnikrishnan M. (2013). Multiple Factors
Modulate Biofilm Formation by the
Anaerobic Pathogen *Clostridium difficile*. J. Bacteriol..

[15] Yamaguchi T., Konishi H., Aoki K., Ishii Y., Chono K., Tateda K. (2020). The Gut Microbiome Diversity of *Clostridioides
difficile*-Inoculated Mice Treated with Vancomycin and Fidaxomicin. J. Infect. Chemother..

[16] Isaac S., Scher J. U., Djukovic A., Jiménez N., Littman D. R., Abramson S. B., Pamer E. G., Ubeda C. (2017). Short- and
Long-Term Effects of Oral Vancomycin on the Human Intestinal Microbiota. J. Antimicrob. Chemother..

[17] Lam S. J., O’Brien-Simpson N. M., Pantarat N., Sulistio A., Wong E. H., Chen Y. Y., Lenzo J. C., Holden J. A., Blencowe A., Reynolds E. C., Qiao G. G. (2016). Combating Multidrug-Resistant
Gram-Negative Bacteria with Structurally Nanoengineered Antimicrobial
Peptide Polymers. Nat. Microbiol..

[18] Shirbin S. J., Insua I., Holden J. A., Lenzo J. C., Reynolds E. C., O’Brien-Simpson N. M., Qiao G. G. (2018). Architectural Effects
of Star-Shaped “Structurally Nanoengineered Antimicrobial Peptide
Polymers” (SNAPPs) on Their Biological Activity. Adv. Healthcare Mater..

[19] Chen Y. F., Lai Y. D., Chang C. H., Tsai Y. C., Tang C. C., Jan J. S. (2019). Star-Shaped Polypeptides
Exhibit Potent Antibacterial
Activities. Nanoscale.

[20] Zheng M., Pan M., Zhang W., Lin H., Wu S., Lu C., Tang S., Liu D., Cai J. (2021). Poly­(α-L-lysine)-Based
Nanomaterials for Versatile Biomedical Applications: Current Advances
and Perspectives. Bioact Mater..

[21] Wilms D., Stiriba S. E., Frey H. (2010). Hyperbranched
Polyglycerols: From
the Controlled Synthesis of Biocompatible Polyether Polyols to Multipurpose
Applications. Acc. Chem. Res..

[22] Phan T. H. M., Yang Y. H., Tsai Y. J., Chung F. Y., Ooya T., Kawasaki S., Jan J. S. (2022). Synthesis
and Hydrogelation of Star-Shaped
Graft Copolypeptides with Asymmetric Topology. Gels.

[23] Ooya T., Ogawa T., Takeuchi T. (2018). Temperature-Induced
Recovery of a
Bioactive Enzyme Using Polyglycerol Dendrimers: Correlation between
Bound Water and Protein Interaction. J. Biomater.
Sci., Polym. Ed..

[24] Laroque S., Locock K. E., Perrier S. (2025). Cationic Star Polymers
Obtained by
the Arm-First Approach—Influence of Arm Number and Positioning
of Cationic Units on Antimicrobial Activity. Biomacromolecules.

[25] Laroque S., Garcia Maset R., Hapeshi A., Burgevin F., Locock K. E., Perrier S. (2023). Synthetic Star Nanoengineered Antimicrobial
Polymers
as Antibiofilm Agents: Bacterial Membrane Disruption and Cell Aggregation. Biomacromolecules.

[26] Chan B. A., Xuan S., Horton M., Zhang D. (2016). 1,1,3,3-Tetramethylguanidine-Promoted
Ring-Opening Polymerization of N-Butyl N-Carboxyanhydride Using Alcohol
Initiators. Macromolecules.

[27] Wang S., Tang Y., Kou X., Chen J., Edgar K. J. (2024). Dextran
Macroinitiator for Synthesis of Polysaccharide-*b*-Polypeptide
Block Copolymers via NCA Ring-Opening Polymerization. Biomacromolecules.

[28] Huang C.-C., Phan T. H. M., Ooya T., Kawasaki S., Lin B.-Y., Jan J.-S. (2022). Effect of Tethered
Sheet-Like Motif and Asymmetric
Topology on Hydrogelation of Star-Shaped Block Copolypeptides. Polymer.

[29] Cheng S., Wang Q., Qi M., Sun W., Wang K., Li W., Lin J., Dong B., Wang L. (2023). Nanomaterials-Mediated
On-Demand and Precise Antibacterial Therapies. Mater. Des..

[30] Lee Y., Sugihara K., Gillilland M. G., Jon S., Kamada N., Moon J. J. (2020). Hyaluronic Acid–Bilirubin
Nanomedicine for Targeted
Modulation of Dysregulated Intestinal Barrier, Microbiome and Immune
Responses in Colitis. Nat. Mater..

[31] Petrey A. C., Obery D. R., Kessler S. P., Zawerton A., Flamion B., de la Motte C. A. (2019). Platelet
Hyaluronidase-2 Regulates the Early Stages
of Inflammatory Disease in Colitis. Blood.

[32] Fin M. T., Diedrich C., Machado C. S., da Silva L. M., Tartari A. P. S., Zittlau I. C., Peczek S. H., Mainardes R. M. (2025). Enhanced
Oral Bioavailability and Biodistribution of Voriconazole through Zein-Pectin-Hyaluronic
Acid Nanoparticles. ACS Appl. Mater. Interfaces.

[33] Yuan L., Wei H., Yang X. Y., Geng W., Peterson B. W., van der
Mei H. C., Busscher H. J. (2021). Escherichia coli Colonization of
Intestinal Epithelial Layers In Vitro in the Presence of Encapsulated *Bifidobacterium breve* for Its Protection against Gastrointestinal
Fluids and Antibiotics. ACS Appl. Mater. Interfaces.

[34] Zhao Y., He Z., Gao H., Tang H., He J., Guo Q., Zhang W., Liu J. (2018). Fine Tuning of Core-Shell Structure
of Hyaluronic Acid/Cell-Penetrating Peptides/siRNA Nanoparticles for
Enhanced Gene Delivery to Macrophages in Antiatherosclerotic Therapy. Biomacromolecules.

[35] Zhao S., Zhao Y., Yang X., Zhao T. (2023). Recent Research Advances
on Oral Colon-Specific Delivery System of Nature Bioactive Components:
A Review. Food Res. Int..

[36] Piotrowski M., Karpiński P., Pituch H., van Belkum A., Obuch-Woszczatyński P. (2017). Antimicrobial Effects of Manuka Honey
on In Vitro Biofilm Formation by *Clostridium difficile*. Eur. J. Clin. Microbiol. Infect. Dis..

[37] Bagdasarian N., Rao K., Malani P. N. (2015). Diagnosis and Treatment of *Clostridium difficile* in Adults: A Systematic Review. JAMA.

[38] Leffler D. A., Lamont J. T. (2015). *Clostridium difficile* Infection. N. Engl. J. Med..

[39] Olsson-Liljequist B., Nord C. E. (2018). Methods for Antimicrobial
Susceptibility Testing of
Anaerobic Bacteria. Clin. Infect. Dis..

[40] Koeth, L. M. Minimum Bactericidal Concentration Testing Clinical Microbiology Procedures Handbook 5th Leber, A. L. ; ASM Press: Washington, DC, 2016; pp. 5.14.1.1–5.14.3.6.

[41] Edwards A. N., McBride S. M. (2016). Isolating and Purifying *Clostridium
difficile* Spores. Methods Mol. Biol..

[42] Weldy M., Evert C., Dosa P. I., Khoruts A., Sadowsky M. J. (2020). Convenient
Protocol for Production and Purification of *Clostridioides
difficile* Spores for Germination Studies. STAR Protoc..

[43] Lee C. C., Tu Y. C., Wu H. T., Ko W. C., Liu H. C., Tsai P. J., Chang H. N., Huang I. H., Hung Y. P. (2025). *Clostridium butyricum* Miyairi Bacteriocin
Treatment for *Clostridioides difficile* Infections
with Clinical Isolates:
Insights from In Vitro, Ex Vivo, and Mouse Model Studies. J. Glob. Antimicrob. Resist..

[44] Rodriguez-Palacios A., LeJeune J. T. (2011). Moist-Heat Resistance, Spore Aging,
and Superdormancy
in *Clostridium difficile*. Appl.
Environ. Microbiol..

[45] Stoltz K. L., Erickson R., Staley C., Weingarden A. R., Romens E., Steer C. J., Khoruts A., Sadowsky M. J., Dosa P. I. (2017). Synthesis and Biological Evaluation
of Bile Acid Analogues
Inhibitory to *Clostridium difficile* Spore Germination. J. Med. Chem..

[46] Bakke R., Kommedal R., Kalvenes S. (2001). Quantification
of Biofilm Accumulation
by an Optical Approach. J. Microbiol Methods.

[47] Hung Y. P., Ko W. C., Chou P. H., Chen Y. H., Lin H. J., Liu Y. H., Tsai H. W., Lee J. C., Tsai P. J. (2015). Proton-Pump
Inhibitor Exposure Aggravates *Clostridium difficile*-Associated Colitis: Evidence from a Mouse Model. J. Infect. Dis..

[48] Malamood M., Nellis E., Ehrlich A. C., Friedenberg F. K. (2015). Vancomycin
Enemas as Adjunctive Therapy for *Clostridium difficile* Infection. J. Clin Med. Res..

[49] Blake S., Thanissery R., Rivera A. J., Hixon M. S., Lin M., Theriot C. M., Janda K. D. (2020). Salicylanilide Analog Minimizes Relapse
of *Clostridioides difficile* Infection in Mice. J. Med. Chem..

[50] Bencherif S. A., Washburn N. R., Matyjaszewski K. (2009). Synthesis
by AGET ATRP of Degradable
Nanogel Precursors for In Situ Formation of Nanostructured Hyaluronic
Acid Hydrogel. Biomacromolecules.

[51] Tashiro S., Taguchi K., Enoki Y., Matsumoto K. (2023). Fecal Pharmacokinetics/Pharmacodynamics
Characteristics of Fidaxomicin and Vancomycin against *Clostridioides
difficile* Infection Elucidated by In Vivo Feces-Based Infectious
Evaluation Models. Clin. Microbiol. Infect..

[52] Pizarro-Guajardo M., Olguín-Araneda V., Barra-Carrasco J., Brito-Silva C., Sarker M. R., Paredes-Sabja D. (2014). Characterization
of the Collagen-Like Exosporium Protein, BclA1, of *Clostridium
difficile* Spores. Anaerobe.

[53] Díaz-González F., Milano M., Olguin-Araneda V., Pizarro-Cerda J., Castro-Córdova P., Tzeng S.-C., Maier C. S., Sarker M. R., Paredes-Sabja D. (2015). Protein Composition of the Outermost
Exosporium-Like Layer of *Clostridium difficile* 630
Spores. J. Proteomics.

[54] Hayashi S., Yoshioka M., Matsui T., Kojima K., Kato M., Kanamaru K., Kobayashi T. (2014). Control of
Reactive Oxygen Species
(ROS) Production through Histidine Kinases in *Aspergillus
nidulans* under Different Growth Conditions. FEBS Open Bio.

[55] Wong J., Chen Y., Gan Y. H. (2015). Host Cytosolic Glutathione
Sensing
by a Membrane Histidine Kinase Activates the Type VI Secretion System
in an Intracellular Bacterium. Cell Host Microbe..

[56] Al
Akeel R., Mateen A., Syed R., Alqahtani M. S., Alqahtani A. S. (2018). Alanine Rich Peptide from *Populus trichocarpa* Inhibit Growth of *Staphylococcus aureus* via Targeting
Its Extracellular Domain of Sensor Histidine Kinase YycGex Protein. Microb. Pathog..

[57] Jones J. B., Liu L., Rank L. A., Wetzel D., Woods E. C., Biok N., Anderson S. E., Lee M. R., Liu R., Huth S. (2021). Cationic Homopolymers Inhibit Spore and Vegetative
Cell Growth of *Clostridioides difficile*. ACS Infect.
Dis..

[58] Janardhanan J., Kim C., Qian Y., Yang J., Meisel J. E., Ding D., Speri E., Schroeder V. A., Wolter W. R., Oliver A. G. (2023). A Dual-Action
Antibiotic that Kills *Clostridioides difficile* Vegetative
Cells and Inhibits Spore Germination. Proc.
Natl. Acad. Sci. U. S. A..

[59] Yoon I. N., Hwang J. S., Lee J. H., Kim H. (2019). The Antimicrobial Peptide
CopA3 Inhibits *Clostridium difficile* Toxin A-Induced
Viability Loss and Apoptosis in Neural Cells. J. Microbiol. Biotechnol..

[60] Paparella A. S., Aboulache B. L., Harijan R. K., Potts K. S., Tyler P. C., Schramm V. L. (2021). Inhibition
of *Clostridium difficile* TcdA and TcdB Toxins with
Transition State Analogues. Nat. Commun..

[61] Sharma S. K., Schilke A. R., Phan J. R., Yip C., Sharma P. V., Abel-Santos E., Firestine S. M. (2023). The Design,
Synthesis, and Inhibition
of *Clostridioides difficile* Spore Germination by
Acyclic and Bicyclic Tertiary Amide Analogs of Cholate. Eur. J. Med. Chem..

[62] Xiao X., Sarma S., Menegatti S., Crook N., Magness S. T., Hall C. K. (2022). In Silico Identification and Experimental Validation
of Peptide-Based Inhibitors Targeting *Clostridium difficile* Toxin A. ACS Chem. Biol..

