# Supplementary material for: Biofilm- and Spore-Disruptive Star-Shaped Poly(l‑lysine)/Hyaluronic Acid Microgels for Targeted Oral Therapy of Clostridioides difficile Infection
Source: Biomacromolecules. 2026 Apr 22;27(5):3019–32. doi: 10.1021/acs.biomac.5c02037 (PMC13169347; doi:10.1021/acs.biomac.5c02037)
Supplement: Supplementary file 1 [file bm5c02037_si_001.pdf]

Supporting Information

Biofilm- and Spore-Disruptive Star-shaped Poly(L-Lysine)/Hyaluronic Acid Microgels for Targeted Oral Therapy of *Clostridioides difficile* Infection

I-Fan Lin, Chia-Yang Chou, Yu-Fang Chen, Ting-Yen Wang, Jing-Ting Lin, Ching-Chi Lee, Jenn-Wei Chen, Jeng-Shiung Jan*, Yuan-Pin Hung*

Corresponding Author

Jeng-Shiung Jan-Department of Chemical Engineering, National Cheng Kung University, No. 1 University Road, Tainan City 701, Taiwan, E-mail: jsjan@mail.ncku.edu.tw, Phone: 886-6-275-7575 ext. 62660, Fax: 886-6-234-4496.

Yuan-Pin Hung, address: Department of Medicine, College of Medicine, National Cheng Kung University, Tainan 70101, Taiwan. Email: yuebin16@yahoo.com.tw, Phone: 886-6-2200055 ext. 3125, Fax: 886-6-2200055 ext. 3125

Authors

I-Fan Lin- Department of Microbiology and Immunology, College of Medicine, National Cheng Kung University, Tainan 70101, Taiwan.

Division of Infectious Diseases, Department of Internal Medicine, E-Da Hospital, I-Shou University, Kaohsiung 82445, Taiwan.

School of Medicine, College of Medicine, I-Shou University, Kaohsiung 82445, Taiwan.

Chia-Yang Chou- Department of Microbiology and Immunology, College of Medicine, National Cheng Kung University, Tainan 70101, Taiwan.

Yu-Fang Chen- Department of Microbiology and Immunology, College of Medicine, National Cheng Kung University, Tainan 70101, Taiwan.

Ting-Yen Wang-Department of Microbiology and Immunology, College of Medicine, National Cheng Kung University, Tainan 70101, Taiwan.

Jing-Ting Lin- Department of Chemical Engineering, National Cheng Kung University, Tainan 70101, Taiwan.

Ching-Chi Lee- Department of Medicine, College of Medicine, National Cheng Kung University, Tainan 70101, Taiwan.

Department of Internal Medicine, College of Medicine, National Cheng Kung University Hospital, National Cheng Kung University, Tainan 70101, Taiwan.

Jenn-Wei Chen- Department of Microbiology and Immunology, College of Medicine, National Cheng Kung University, Tainan 70101, Taiwan.

Supporting information for Material and Methods

Synthesis of ZLL NCA

ZLL NCA was synthesized according to the Fuchs-Farthing method using triphosgene. Briefly, H-Lys(Z)-OH (1.0 eq.) and triphosgene (0.33 eq.) were weighed and transferred into a round-bottom flask within a nitrogen-filled glovebox. Anhydrous tetrahydrofuran (THF) was added to the monomer and triphosgene at a volume 10 times the weight of each reagent (10 mL g^{-1}). Once the triphosgene was completely dissolved, it was added to the H-Lys(Z)-OH suspension. The flask was sealed, and stirred in an oil bath at 55 °C.

The reaction was completed after 1–2 h when the mixture became a clear, transparent solution. The resulting ZLL NCA solution was then transferred via a cannula into a 5-fold volume of chilled n-hexane for precipitation. The mixture was kept at -20 °C overnight. The supernatant was subsequently removed through a filter-tipped cannula. Residual solvents were eliminated using a high-vacuum pump until the product was completely dry, yielding the final product as a fine white powder (yield: ~90%).

Synthesis of Star-shaped Polypeptides (G2-PLL and G3-PLL)

The star-shaped protected polypeptides were synthesized via ring-opening polymerization (ROP). In a nitrogen-filled glovebox, the initiators (PGD-G2 or PGD-G3) were dissolved in anhydrous DMF to a final concentration of 0.02 M. To activate the hydroxyl terminal groups of the initiators, 1,1,3,3-tetramethylguanidine (TMG) was added to the solution at a concentration of 6 mM and stirred for 30 min. Subsequently, the ZLL NCA monomer, previously prepared as a 1 mM solution in DMF, was added to the mixture. The reaction flask was sealed and stirred at room temperature for 48 h.

Upon completion, the reaction mixture was transferred into a dialysis bag (MWCO: 12–14 kDa). The crude product was first dialyzed against DMF for 1 hour, followed by dialysis against methanol for 4 h. Finally, the solution was dialyzed against double-distilled water (ddH_2O) for 3–5 days. The protected polymers, G2-PZLL and G3-PZLL, were obtained as solid products after lyophilization.

To obtain the final star-shaped poly(L-lysine), the benzyloxycarbonyl (Z) protective groups were removed. The G2-PZLL or G3-PZLL was dissolved in trifluoroacetic acid at a 50-fold volume relative to the polymer weight. Hydrogen bromide (HBr, 33 wt% in acetic acid) was added at a 5-fold molar excess relative to the Z-groups, and the reaction was maintained for 30 minutes to facilitate the cleavage of the protective groups.

The deprotected polymer was precipitated by pouring the reaction mixture into diethyl ether. The solid product was collected by centrifugation at 6000 rpm for 10 min. The obtained solid was redissolved in ddH_2O, and the pH was adjusted to 6.0–8.0 using a sodium hydroxide (NaOH) solution. The solution was then dialyzed against ddH_2O for 3 to 5 days. The final star-shaped poly(L-lysine) was obtained as a white powder after lyophilization.

Culture media for Clostridioides difficile

C. difficile bacteria were grown on CDC anaerobic agar plates supplemented with 5% sheep blood or in a Brain Heart Infusion Supplement (BHIS) medium containing 5 mg mL^{-1} yeast extract and 0.1% L-cysteine. The cultures were incubated at 37°C in an anaerobic chamber (Whitley A20 Workstation, Don Whitley Scientific, UK), which provided a stable atmosphere containing a gas mixture (5% CO_2, 10% H_2, 85% N_2) [1].

Antimicrobial susceptibility testing of G3-PLL$_9$

To determine the minimum inhibitory concentration (MIC) and minimum bactericidal concentration (MBC) of G3-PLL$_9$ against *C. difficile*, bacteria were cultured anaerobically on CDC agar plates at 37°C for 48 h. Colonies with consistent morphology were collected and adjusted to 0.5 McFarland density (representing 1.5×10^8 colony-forming unit [CFU] mL^{-1}) using a densitometer, then diluted 100-fold with BHIS medium. G3-PLL$_9$ stock solutions were serially diluted in BHIS to concentrations ranging from 0.25 to 32 μM.

The experimental setup involved combining 1 mL of the diluted bacterial suspension with varying concentrations of 1 mL G3-PLL$_9$ in 3 mL test tubes. Controls consisted of 1 mL of 1 μM vancomycin and 1 mL of BHIS medium. Quality control was conducted using *C. difficile* ATCC® 700057 treated with vancomycin concentrations ranging from 0.25 to 2 mg L^{-1}. Initial bacterial counts were determined by plating onto CDC agar plates after serial dilution.

Anaerobic incubation of the *C. difficile* broth at 37°C for 48 h facilitated visual inspection for turbidity. The MIC was established as the minimum concentration at which the broth remained clear after 48 h. For MBC determination, 100-μL aliquots were extracted from broth concentrations at or above the MIC. These aliquots were serially diluted and plated onto CDC agar plates to identify the minimum concentrations that reduced colony counts to less than 0.1% of the initial test inoculum after 48 h of incubation [2].

Before the time-kill analysis, *C. difficile* colonies on CDC agar plates were transferred to BHIS medium and incubated anaerobically at 37°C for 12–16 h to reach the logarithmic growth phase, verified by optical density (OD) measurement [3]. Following this, the bacterial suspension

was adjusted to 0.5 McFarland density, combined with various concentrations of G3-PLL_9, and incubated anaerobically at 37°C. Bacterial counts of *C. difficile* inoculum were measured at intervals of 0, 4, 8, 12, 24, and 48 h by withdrawing 100-µL aliquots for serial dilution and plating onto CDC agar plates to create a time-kill curve.

Serial passage resistance assay

To assess the potential for resistance development under prolonged sub-inhibitory exposure, a serial passage assay was conducted. Following initial MIC determination, cultures grown in 0.5× MIC were adjusted to a 0.5 McFarland standard, diluted 100-fold in BHIS medium, and 100 µL aliquots were mixed with 100 µL of G3-PLL_9 (ranging from 0.25× to 4× MIC) in pre-loaded 96-well plates. MICs were determined as before, based on visual broth clarity after 48 h of anaerobic incubation. Cultures grown in 0.5× MIC were used to initiate the next passage. This procedure was repeated for ten consecutive passages.

C. difficile spore preparation and purification

C. difficile was cultured anaerobically on CDC agar plates at 37°C for 48 h and adjusted to an OD_{600} value of 0.2 with sterile water. Next, 990 µL of the bacterial suspension was placed onto a 9-cm dish containing 70:30 Sporulation Medium [4, 5]. The medium consisted of 63 mg mL^{-1} Bacto peptone (trypsin), 3.5 mg mL^{-1} Proteose-peptone, 0.7 mg mL^{-1} ammonium sulfate, 1.06 mg mL^{-1} Tris base, 11.1 mg mL^{-1} BHI, 1.5 mg mL^{-1} yeast extract, 0.3 mg mL^{-1} L-cysteine, and 15 mg mL^{-1} bacterial agar. The dish was incubated anaerobically at 37°C for 7–10 days to induce sporulation.

After incubation, spores were harvested from the medium using sterile ice-cold water and stored overnight at 4 °C to facilitate spore release from the mother cell [5]. The following day, the spores were washed five times with sterile ice-cold water and centrifuged at 4000 × g for 10 min. The pellet was resuspended in 200 µL of sterile ice-cold water and underwent equilibrium density gradient centrifugation using 1 mL of 50% (wt/vol) Nycodenz® at 10800 × g for 1 h to separate spores from vegetative cells [6]. The upper Nycodenz® layer and cell debris were discarded, and the spores were washed five times with sterile ice-cold water to remove Nycodenz®. Finally, the purified spores were stored at 4 °C in the dark.

G3-PLL_9 sporicidal analysis

The purified *C. difficile* spores underwent a sublethal heat treatment at 60°C using a dry bath for 30 min. This procedure aimed to diminish the vegetative cells of germinated spores in the stock while reactivating dormant spores [7]. To analyze the spore viability, they were adjusted

to an OD_{600} of 0.2, approximately 1 × 107 CFU mL^{-1}, a common concentration for spore property testing [8]. Subsequently, the spores were treated with G3-PLL_9 at concentrations ranging from 1 to 16 μM for 30 min, followed by supplementation with 10 mM taurocholic acid for 12 min 6. Non-germination controls were established by omitting the taurocholic acid. The mixture was serially diluted with 1× phosphate buffered saline (PBS) and plated on CDC agar plates, which were then anaerobically incubated at 37°C for 48 h before CFU enumeration.

Spore germination assay

G3-PLL_9 was studied for its ability to inhibit *C. difficile* spore germination. Preheated spores were exposed to G3-PLL_9 for 30 min, followed by germination induction. The germination process was monitored by measuring OD600 every minute using a microplate reader (SpectraMax® i3x Multi-Mode Detection Platform). The extent of germination was determined by calculating the percentage decrease in OD600 from the initial value [9].

Bacterial biofilm preparation and quantification

C. difficile biofilms were generated by inoculating *C. difficile* broth into a 96-well flat-bottomed microplate as previously reported [10]. First, 2 mL of 0.5-McFarland *C. difficile* overnight growth broth was diluted with 18 mL of BHIS (0.1×). Next, 200 μL aliquots were added to each well and incubated anaerobically at 37°C for 48 h. Negative control wells contained BHIS only. Subsequently, the medium was removed, and wells were washed twice with PBS and air-dried at 37°C for 15 min. G3-PLL_9 concentrations ranging from 0.25 to 8 μM were added in triplicates, while 1 μM vancomycin was added as control and PBS for the negative control. The microplate was then incubated at 37°C for 48 h. After incubation, the supernatants were removed, and the remaining biofilms were stained with 50 μL crystal violet for 10 min. Following this, wells were washed eight times with PBS and air-dried at 37°C for 15 min. The remaining crystal violet within the biomass was dissolved in 100 μL ethanol, and the OD_{570} absorbance was measured using a microplate reader (SpectraMax® i3x Multi-Mode Detection Platform).

Conjugation of amine dye with G3-PLL_9

To enhance the specificity of G3-PLL_9 visualization under fluorescence microscopy, an amine-reactive dye was conjugated to the polypeptide before co-treatment with *C. difficile* spores. G3-PLL_9 (10 mg) was dissolved in 1 mL of 0.1 M sodium bicarbonate buffer and adjusted to pH 8.3. Then, an amine-reactive dye (Alexa Fluor® 350, Thermo Fisher Scientific) was dissolved in anhydrous DMF at a concentration of 10 mg mL^{-1}. While gently stirring the

protein solution, 50–100 µL of the reactive dye solution was gradually added to facilitate the conjugation reaction. The mixture was incubated for 1 h at room temperature with continuous stirring. After incubation, the mixture was dialyzed using dry dialysis tubing (Spectra/Por 3, 3.5 kDa) to eliminate any unreacted dye and other impurities. The amine-conjugated G3-PLL_9 was then cryodesiccated and stored at −20°C until use.

Fluorescence microscopy and quantification

C. difficile spores at an OD_{600} of 1.0 were treated with either G3-PLL_9 or PBS for 10 min, then stained using the Live/DeadTM BacLightTM Bacterial Viability Kit (Thermo Fisher Scientific) according to the manufacturer's instructions. The green-fluorescent nucleic acid stain SYTO-9 was used to label both viable and non-viable spores. Propidium iodide (PI), a red-fluorescent nucleic acid stain, selectively labeled non-viable spores after outer layer damage, allowing PI penetration. A 10-µL drop of the stained spores was placed on slides, covered with a coverslip, and observed through an upright fluorescent microscope (Olympus, BX53). On average, ten images were captured per experiment group. Dead cells, indicated by yellow or white coloration (when amine dye was employed) in combined-layer images, were enumerated and compared with the untreated group using ImageJ software developed by the National Institutes of Health. The proportion of viable and non-viable cells was calculated as the respective cell count divided by the total cell count (viable + non-viable) and expressed as a percentage.

ATP-based bacterial viability assay

The effect of G3-PLL_9 on *C. difficile* cell viability was assessed using the BacTiter-Glo™ Microbial Cell Viability Assay (Promega, Cat# G8230). *C. difficile* strain JIK 8284 was subcultured overnight, harvested, and resuspended in PBS to a final density of 1×10^4 cells per 50 µL. In an opaque-walled 96-well plate, 50 µL of drug solution was added per well, followed by 50 µL of bacterial suspension (final volume: 100 µL/well). G3-PLL_9 was tested at final concentrations of 16, 8, 4, 2, and 1 µM by two-fold serial dilution in PBS. Vancomycin (final concentration: 1× MIC) and fidaxomicin (final concentration: 1× MIC) served as antibiotic controls. Negative controls received 50 µL PBS in place of drug, and blank controls contained 100 µL PBS without bacteria. All conditions were tested in triplicate. After 30 minutes of incubation, 100 µL of BacTiter-Glo™ reagent was added to each well at a 1:1 ratio, mixed briefly, and incubated for 5 minutes at room temperature. Luminescence was recorded using a microplate luminometer. Luminescence intensity reflects intracellular ATP content and is proportional to the number of viable bacterial cells.

RNA sequencing and differential expression gene analysis

RNA sequencing was performed to investigate G3-PLL$_9$'s antimicrobial mechanism against *C. difficile*. Log-phase bacterial cultures (10^8 CFU mL^{-1}) were treated with 4 µM G3-PLL$_9$ for 15 min, with untreated samples serving as controls. After centrifugation, cell pellets were preserved in DNA/RNA Shield (Zymo Research) and stored at -80 °C. Total RNA was extracted and quality-checked using NanoDrop and Fragment Analyzer 5200. Sequencing was performed on Illumina NovaSeq X Series platform at Genomics, Taiwan. Data quality was assessed using FastQC v0.12.0, and analysis followed the ProkSeq workflow using *C. difficile* strain 630 delta erm as reference. Reads were aligned using Bowtie2, and gene quantification was performed using FeatureCounts. Differential expression analysis used NOISeq algorithm, with significant genes defined as $|\log2(\mathrm{FoldChange})| > 1$ and probability ≥ 0.8. These genes were further analyzed using Gene Ontology (GO) and Kyoto Encyclopedia of Genes and Genomes (KEGG) pathway enrichment analysis.

Hemolysis assay

The hemolytic activity of G3-PLL$_9$ was assessed following a previously described protocol [11]. Briefly, red blood cells were washed three times with PBS (0.1 M, pH 7.2) and centrifuged at 1000 × g for 10 min. A 100 µL aliquot of the cell suspension was diluted to a final concentration of 5% (v/v) in PBS and added into a 96-well plate. G3-PLL$_9$ solutions, prepared by twofold serial dilutions ranging from 0.5 µM to 64 µM, were then added (100 µL per well) to the plate. After incubating the mixtures at 37 °C for 1 h, the plates were centrifuged at 1000 × g for 10 min. Supernatants (100 µL) were transferred to a new 96-well plate, and the absorbance at 540 nm was measured to quantify released hemoglobin. A 0.1% (v/v) Triton X-100 treatment was used as a positive control to define complete hemolysis (100%), while PBS served as a negative control. Vancomycin (0.5–64 µM) was included as an additional comparator. Hemolytic activity (%) was calculated using the formula: [(Asample – APBS) / (ATriton – APBS)] × 100, where Asample is the absorbance of wells treated with G3-PLL$_9$ or vancomycin, APBS represents the negative control with PBS, and ATriton represents the positive control with Triton X-100.

Animals and C. difficile infection (CDI) mouse model

Male C57BL/6JNral mice aged six to eight weeks were purchased from the National Laboratory Animal Center in Taiwan. The mice were housed in groups of five and provided with autoclaved food and water at the Laboratory Animal Center of the College of Medicine at NCKU. Housing conditions included a 13-hour light/11-hour dark cycle (7AM–8PM),

temperatures ranging from 23 ± 1 °C, and humidity levels of 45 ± 15%. All animal-related studies were performed following a protocol approved by the Institutional Animal Care and Use Committee of NCKU (IACUC Approval No.: 112234).

To disrupt the intestinal microbiota and establish the CDI model, seven-week-old mice received a cocktail of antibiotics (vancomycin, 0.045 mg mL^{-1}; metronidazole, 0.215 mg mL^{-1}; kanamycin, 0.4 mg mL^{-1}; colistin, 0.057 mg mL^{-1}; and gentamicin, 0.035 mg mL^{-1}) in their drinking water daily for five days. Metronidazole and vancomycin were removed from the drinking water one day before infection to avoid interference with *C. difficile* colonization.

On the day of infection, mice received clindamycin intraperitoneally (4 mg kg^{-1}) and oral dose of proton-pump inhibitor esomeprazole (Nexium®, 0.1 mg) [12]. Mice were then inoculated orally with *C. difficile* spores (106 CFU per 100 µL) [12]. To prevent degradation by gastric acidity, G3-PLL$_9$ was delivered via anal enema. Mice received 30-µL enemas containing G3-PLL$_9$ at concentrations of 4, 1, and 0.25 µM, or vancomycin at 50 mg kg^{-1} day^{-1}, administered at 8, 24, and 48 h post-infection [13]. For hyaluronic acid conjugated G3-PLL$_9$, 100 µL of which 5, 2.5, 1 µM was administered orally following the same timing protocol used for the enemas.

In the primary infection model, mice were euthanized 52 h post-infection, while in the recurrent CDI model, they were observed for up to two weeks post-infection. Blood specimens were collected and centrifuged at 3000 rpm for 30 min to obtain serum samples for biochemical tests. These samples were then stored at −20 °C. Colon length and cecum weight were measured, and specimens were longitudinally excised from both the upper (cecum) and lower (rectum) ends of the colon for further histopathological examination. Additionally, stool samples were collected for DNA extraction.

Hyaluronidase quantification

Hyaluronidase activity in the mouse colon was quantified using a commercially available Enzyme-Linked Immunosorbent Assay (ELISA) kit performed in a 96-well plate. Colonic mucus was collected by scraping the colon 30 times using a sterile loop and diluted in PBS. A seven-point standard curve was prepared by serial twofold dilutions of a 5000 pg mL^{-1} stock solution.

For the assay, 100 µL of each standard, blank, and samples were added to the respective wells, covered with a plate sealer, and incubated at 37 °C for 1 h. After incubation, the liquid was aspirated, and 100 µL of Detection Reagent A was added to each well. The plate was re-sealed and incubated for an additional hour at 37 °C. Following incubation, the wells were

washed three times with 300 μL of Wash Solution. Next, 100 μL of Detection Reagent B was added to each well, covered, and incubated at 37 °C for 30 min. Then the wash process was repeated five times. Subsequently, 90 μL of 3,3',5,5'-Tetramethylbenzidine Substrate Solution was added to each well and incubated at room temperature, protected from light, for 5–10 min. During this step, the solution gradually turned blue. Lastly, the reaction was halted by adding 50 μL of Stop Solution, turning the solution yellow. Finally, OD was measured at 450 nm using a Multiskan Sky Microplate Spectrophotometer (Thermo Scientific).

Stool DNA extraction

The collected stools from euthanized mice were preserved in 500 μL of DNA/RNA Shield (Pangea Laboratory) at −20°C. DNA was extracted from stool samples using a homogenizer (MagNA Lyser Instrument, Roche Applied Science) followed by the high-pure PCR template preparation kit (Roche) protocol, based on affinity chromatography. The extracted DNA was eluted using centrifugation with 50 μL of diethylpyrocarbonate (DEPC) water and then stored at −20 °C. DNA concentrations were evaluated using a NanoDrop 2000 spectrophotometer.

Real-time polymerase chain reaction (PCR)

Each 20-μL PCR reaction volume contained 10 μL of 2× PowerSYBR® Green PCR Master Mix (Applied Biosystems), 0.5 μL each of 20 μM forward and reverse primers (tcdBF, 5’-GGTATTACCTAATGCTCCAAATAG-3’; tcdBR, 5’-TTTGTGCCATCATTTTCTAAGC-3’) [14], and 5 μL of stool DNA sample dissolved in DEPC water at a concentration of 0.8 ng μL^{-1}. The fluorescence signal was monitored using a StepOnePlus™ (Applied Biosystems) thermal cycler programmed with an initial step at 95°C for 10 min, followed by 40 cycles of denaturation at 95°C for 15 seconds and annealing/extension at 60°C for one minute. A melting curve analysis was then conducted: 95 °C for 15 seconds, 60 °C for 1 minute, and a final step at 95 °C for 15 seconds. The relative signal of *C. difficile tcdB* gene was normalized to the signal from the universal primers (926F, 5’-AAACTCAAAKGAATTGACGG-3’; 1062R, 5’-CTCACRRCACGAGCTGAC-3’) [15], and further analysis was conducted using the $2^{-\Delta\Delta ct}$ method [16].

Bacterial 16S rRNA sequencing and bioinformatics analysis

The bacterial V3–V4 region of 16S rRNA was amplified using the specific primer pair 314F (5′-TCGTCGGCAGCGTCAGATGTGTATAAGAGACAGCCTACGGGNGGCWGCAG-3′) and 805R (5′-

GTCTCGTGGGCTCGGAGATGTGTATAAGAGACAGGACTACHVGGGTATCTAATCC-3′). The total reaction volume was 25 μL, containing 2.5 μL DNA template, 5 μL of each primer, and 12.5 μL KAPA HiFi HotStart ReadyMix (Roche Sequencing Solutions, Pleasanton, CA, USA [KK2601]) to make up the final volume. The reactions were implemented through the following steps: 95 °C for 5 min; followed by 30 cycles of 95 °C for 30 s, 60 °C for 30 s, 72 °C for 30 s; and final extension at 72 °C for 5 min. The PCR products were stored at 4 °C and then used as templates for Index PCR in a total 25 μL volume, including 2.5 μL PCR template, 2.5 μL of each Nextera XT Index primer, 12.5 μL KAPA HiFi HotStart ReadyMix, and 5 μL ddH_2O. The Index PCR was run under the following conditions: 95 °C for 30 s; 8 cycles of 95 °C for 30 s, 60 °C for 30 s, and 72 °C for 30 s; and final extension at 72 °C for 5 min. DNA samples were paired-end sequenced (2 × 300 bp) on an Illumina MiSeq platform (Illumina, San Diego, USA) by Majorbio Bio-Pharm Technology Co., Ltd. (Shanghai, China). Sequence data were performed using 16S Metagenomics apps on Basespace (Illumina, San Diego, CA, USA) and the reads were clustered to the operational taxonomic unit (OTU) with the Illumina-curated version of the May 2013 Greengenes taxonomic database. The downstream analysis was performed using the CLC Microbial Genomics Module (v24.0, Qiagen, Germany). The alpha diversity was measured using the Shannon index, and beta diversity was measured using Bray–Curtis. Permutational multivariate analysis of variance (PERMANOVA) was used to analyze statistical differences in beta diversity. Statistical significance was set at $p < 0.05$. Hierarchical clustering of the top 25 abundant taxonomies was deduced using a heatmap to determine patterns between groups. An OTU table was generated by the CLC Microbial Genomics Module. Linear discriminant analysis Effect Size (LEfSe) was performed using QIIME2 version 2023.2 (https://qiime2.org), with an alpha value of 0.05 and an LDA score cut-off of 2.0. Functional predictions were made using Phylogenetic Investigation of Communities by Reconstruction of Unobserved States (PICRUSt) [17].

Histopathological examination

The collected colon samples were fixed in 4% formaldehyde solution buffered with PBS and then embedded in paraffin. Sections of 5 μm thickness were prepared and deparaffinized for subsequent staining with hematoxylin and eosin. The process of tissue embedding, sectioning, deparaffinization, and staining was conducted by the Human Biobank, Research Center of Clinical Medicine in NCKU Hospital. These stained sections were observed under an optical microscope (Olympus BX61) with image capture facilitated by CellSens software (Olympus®).

For histological injury scoring, six high-power fields were selected from each sample. Scoring was based on the assessment of epithelial tissue damage, mucosal edema, and neutrophil infiltration. Each category received a score ranging from 0 to 3, with 0 indicating no observed pathology. The individual scores for each category were summed to calculate an overall disease severity score [18].

Table S1. The feed molar ratio for the polypeptide synthesis, and the degree of polymerization (DP), number-average molecular weight (M_n), and molecular weight distribution (M_w / M_n) derived from NMR and GPC-LS analyses.

Polypeptide	Feed molar ratio	^{1}H NMR		GPC-LS		
	Initiator : ZLL NCA	DP	M_n	M_n	M_w / M_n	DP
G_2-$PZLL_{10}$	1 ∶ 120	10	32200	32100	1.6	10
G_3-$PZLL_9$	1 ∶ 240	10	64000	57000	1.5	9

NMR, nuclear magnetic resonance. GPC-LS, gel permeation chromatography-light scattering. ZLL NCA, Z-L-Lysine N-carboxyanhydride.

Table S2. Minimum bactericidal concentrations (MBCs) of G3-PLL_9 against various bacterial isolates.

Bacteria	Minimum bactericidal concentration (μM)	
	G3-PLL_9	Vancomycin
Staphylococcus aureus[1]	16	-
Escherichia coli[1]	64	-
Klebsiella pneumoniae[1]	> 64	-
Clostridioides difficile		
Vancomycin-resistant[2]	16	> 64
Metronidazole-resistant[2]	16	8
Ribotype 017[2]	8	≤ 1
Ribotype 027	8	≤ 1

[1]Standard ATCC strains.

[2]Clinical isolates: The vancomycin-resistant clinical isolate (CMMC-42; ribotype 078, *tcdC*$^{-}$) was obtained from Chi Mei Medical Center, Taiwan. The metronidazole-resistant clinical isolate (NCKU-I2) was obtained from National Cheng Kung University Hospital, Taiwan. The ribotype 017 clinical isolate (TNHP-235) was obtained from Tainan Hospital, Taiwan.

Table S3. List of 45 differentially expressed genes and their normalized gene expression level.

Gene ID	G3-PLL$_9$	Untreated	M (log$_2$FC)	D	Prob	Ranking	Gene symbol	Gene description	Gene ID (latest)
CDIF630erm_02824	206.8807391	2.184843449	6.565125614	204.6958956	0.948109568	204.8011488		response regulator transcription factor	CDIF630erm_RS14075
CDIF630erm_02823	409.1697995	13.85404717	4.884320268	395.3157523	0.924864969	395.3459252		sensor histidine kinase	CDIF630erm_RS14070
CDIF630erm_03452	195.0912682	18.07273009	3.432262845	177.0185381	0.85773534	177.0518096		ABC transporter permease	CDIF630erm_RS17195
CDIF630erm_00019	5880805.157	859884.8936	2.773798237	5020920.263	0.897762346	5020920.263		23S ribosomal RNA	CDIF630erm_RS00095
CDIF630erm_00013	5819264.424	864372.2038	2.751112218	4954892.22	0.897472994	4954892.22		23S ribosomal RNA	CDIF630erm_RS00065
CDIF630erm_00037	1623.821487	241.8956791	2.746936137	1381.925808	0.877989969	1381.928538	*rrf*	5S ribosomal RNA	CDIF630erm_RS00170
CDIF630erm_00036	5715873.03	871107.5049	2.714051189	4844765.525	0.896749614	4844765.525		23S ribosomal RNA	CDIF630erm_RS00165
CDIF630erm_00208	5851782.761	902495.3158	2.696884863	4949287.445	0.896460262	4949287.445		23S ribosomal RNA	CDIF630erm_RS01010
CDIF630erm_002	8299.47517	1362.94745	2.60629016	6936.52772	0.88618827	6936.52821	*rrf*	5S ribosomal	CDIF630erm_RS010

09	2	1	9	1	2	1		RNA	15
CDIF630erm_00020	8357.577845	1381.77741	2.596559657	6975.800436	0.886188272	6975.800919	*rrf*	5S ribosomal RNA	CDIF630erm_RS00100
CDIF630erm_00441	8661.933869	1452.78659	2.575866367	7209.147279	0.886236497	7209.147739	*rrf*	5S ribosomal RNA	CDIF630erm_RS02175
CDIF630erm_00215	7449.765526	1301.233168	2.517315517	6148.532358	0.885561343	6148.532873	*rrf*	5S ribosomal RNA	CDIF630erm_RS01045
CDIF630erm_00014	7796.197869	1372.484903	2.505980426	6423.712965	0.885561343	6423.713454	*rrf*	5S ribosomal RNA	CDIF630erm_RS00070
CDIF630erm_01174	8753.494922	1572.154077	2.477116529	7181.340846	0.883921682	7181.341273	*rrf*	5S ribosomal RNA	CDIF630erm_RS06035
CDIF630erm_00799	116.4865094	21.30846962	2.450663996	95.17803982	0.808449074	95.2095847	*rpmI*	50S ribosomal protein L35	CDIF630erm_RS04200
CDIF630erm_03869	7250865.949	1330041.862	2.446681648	5920824.087	0.892071759	5920824.087		23S ribosomal RNA	CDIF630erm_RS19510
CDIF630erm_00202	7706.323269	1415.243578	2.444992329	6291.07969	0.882908951	6291.080165	*rrf*	5S ribosomal RNA	CDIF630erm_RS00980
CDIF630erm_02979	7251202.542	1366862.076	2.407352598	5884340.466	0.891782407	5884340.466		23S ribosomal RNA	CDIF630erm_RS14825
CDIF630erm_03868	10736.25098	2036.670115	2.398206076	8699.58087	0.883198302	8699.5812	*rrf*	5S ribosomal RNA	CDIF630erm_RS19505

CDIF630erm_03723	10040.9727	1995.688325	2.330940703	8045.284373	0.882426698	8045.284711	*rrf*	5S ribosomal RNA	CDIF630erm_RS18785
CDIF630erm_01767	190.0243494	40.71255464	2.222638639	149.3117948	0.827787423	149.3283368	*cysK*	cysteine synthase A	CDIF630erm_RS09030
CDIF630erm_03725	1401467.135	303248.075	2.208367521	1098219.06	0.885706019	1098219.06		16S ribosomal RNA	CDIF630erm_RS18795
CDIF630erm_02980	1398678.884	303172.7252	2.205852906	1095506.159	0.885706019	1095506.159		16S ribosomal RNA	CDIF630erm_RS14830
CDIF630erm_03870	1405753.565	306598.9331	2.196919123	1099154.631	0.885561343	1099154.631		16S ribosomal RNA	CDIF630erm_RS19515
CDIF630erm_00012	1273885.718	288348.6825	2.143349521	985537.0351	0.881896219	985537.0351		16S ribosomal RNA	CDIF630erm_RS00060
CDIF630erm_00177	1254638.605	288862.9692	2.118814686	965775.636	0.880111883	965775.6361		16S ribosomal RNA	CDIF630erm_RS00855
CDIF630erm_00201	7790977.85	1806465.181	2.108634965	5984512.669	0.881510417	5984512.669		23S ribosomal RNA	CDIF630erm_RS00975
CDIF630erm_00180	6375.394437	1486.142783	2.100941871	4889.251653	0.871431327	4889.252105	*rrf*	5S ribosomal RNA	CDIF630erm_RS00870
CDIF630erm_00199	1295499.275	302127.406	2.100279245	993371.8685	0.879774306	993371.8685		16S ribosomal RNA	CDIF630erm_RS00965
CDIF630erm_00017	1316797.342	308048.1198	2.095805693	1008749.222	0.879822531	1008749.222		16S ribosomal RNA	CDIF630erm_RS00085

CDIF630erm_00214	7831707.145	1845110.98	2.085619224	5986596.165	0.880642361	5986596.165		23S ribosomal RNA	CDIF630erm_RS01040
CDIF630erm_00212	1304811.62	311114.8854	2.068322207	993696.7346	0.877555941	993696.7346		16S ribosomal RNA	CDIF630erm_RS01030
CDIF630erm_00439	1337696.118	319252.9047	2.066978768	1018443.214	0.877604167	1018443.214		16S ribosomal RNA	CDIF630erm_RS02165
CDIF630erm_01171	1155064.03	277805.6351	2.055825059	877258.3945	0.877218364	877258.3945		16S ribosomal RNA	CDIF630erm_RS06020
CDIF630erm_01172	7232907.09	1744551.999	2.05171902	5488355.09	0.87890625	5488355.09		23S ribosomal RNA	CDIF630erm_RS06025
CDIF630erm_00035	1150058.948	278635.0266	2.045259278	871423.9214	0.877121914	871423.9214		16S ribosomal RNA	CDIF630erm_RS00160
CDIF630erm_00179	7492371.631	1840068.978	2.025662612	5652302.653	0.878568673	5652302.653		23S ribosomal RNA	CDIF630erm_RS00865
CDIF630erm_03724	10046064.58	2526816.514	1.991237643	7519248.066	0.876109182	7519248.066		23S ribosomal RNA	CDIF630erm_RS18790
CDIF630erm_00440	6838750.284	1729459.643	1.983411362	5109290.641	0.876060957	5109290.641		23S ribosomal RNA	CDIF630erm_RS02170
CDIF630erm_00206	855299.2246	218898.632	1.966166293	636400.5925	0.87345679	636400.5925		16S ribosomal RNA	CDIF630erm_RS01000
CDIF630erm_00423	253.8800094	66.06648081	1.942156398	187.8135286	0.825327932	187.8235701		LacI family DNA-binding	CDIF630erm_RS02080

								transcriptional regulator	
CDIF630erm_01840	182.8201802	50.27298342	1.862570115	132.5471967	0.807243441	132.5602826		HAD-IC family P-type ATPase	CDIF630erm_RS09365
CDIF630erm_00028	466863.0243	177513.8298	1.395067904	289349.1945	0.830777392	289349.1945	*ffs*	signal recognition particle sRNA large type	CDIF630erm_RS00130
CDIF630erm_01878	8799.780869	3431.174371	1.358765153	5368.606497	0.820264275	5368.606669		NifB/NifX family molybdenum-iron cluster-binding protein	CDIF630erm_RS09565
CDIF630erm_01852	1691.339771	5272.132742	-1.640220185	3580.792971	0.843267747	-3580.793347		hypothetical protein	CDIF630erm_RS20670

M, log2 fold change (FC) in gene expression levels. D, the absolute difference in expression levels between two treatment conditions. Prob, probability of differential expression. Ranking: summary statistic incorporating M and D values.

Table S4. Enrichment analysis of differentially expressed genes.

Gene set	Description	GeneRatio	BgRatio	pvalue	p.adjust	qvalue	GeneID	Count
Kyoto Encyclopedia of Genes and Genomes (KEGG)								
KEGG:00920	Sulfur metabolism	1/1	5/455	0.010989	0.021978	0.011567	CDIF630erm_01767	1
KEGG:00270	Cysteine and methionine metabolism	1/1	24/455	0.052747	0.052747	0.027762	CDIF630erm_01767	1
Gene ontology (GO)								
GO:0007165	signal transduction	1/6	41/2326	0.101313	0.191327	0.191327	CDIF630erm_02823	1
GO:0000155	phosphorelay sensor kinase activity	1/6	43/2326	0.106027	0.191327	0.191327	CDIF630erm_02823	1
GO:0000166	nucleotide binding	1/6	48/2326	0.117723	0.191327	0.191327	CDIF630erm_01840	1
GO:0005840	ribosome	1/6	48/2326	0.117723	0.191327	0.191327	CDIF630erm_00799	1
GO:0003735	structural constituent of ribosome	1/6	52/2326	0.126988	0.191327	0.191327	CDIF630erm_00799	1
GO:0006355	regulation of transcription, DNA-templated	2/6	250/2326	0.129081	0.191327	0.191327	CDIF630erm_00423/ CDIF630erm_02824	2
GO:0006412	translation	1/6	54/2326	0.13159	0.191327	0.191327	CDIF630erm_00799	1
GO:0005622	intracellular	1/6	58/2326	0.140733	0.191327	0.191327	CDIF630erm_00799	1
GO:0000160	phosphorelay signal transduction system	1/6	65/2326	0.15654	0.191327	0.191327	CDIF630erm_02824	1

GO:0003677	DNA binding	2/6	327/2326	0.201471	0.221618	0.221618	CDIF630erm_00423/ CDIF630erm_02824	2
GO:0016021	integral component of membrane	1/6	185/2326	0.392146	0.392146	0.392146	CDIF630erm_01840	1

GeneRatio, the number of genes annotated in the specific gene set. BgRatio, the ratio of the number of genes in the background set that are annotated in the specific gene set to the total number of genes in the background set. pvalue: p-value, the statistical significance of the enrichment. p.adjust, the p-value adjusted for multiple comparisons using the Benjamini–Hochberg method to control the False Discovery Rate (FDR). qvalue, q-value estimation for a given set of p-values considering the FDR. Count, the number of genes from the sample annotated in the specific gene set.

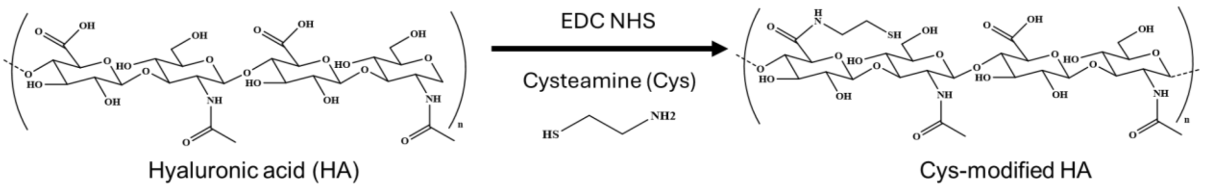

Figure S1. Synthesis scheme of cysteamine-modified hyaluronic acid.

EDC, 1-ethyl-3-(3-dimethylaminopropyl) carbodiimide. NHS, N-hydroxysuccinimide.

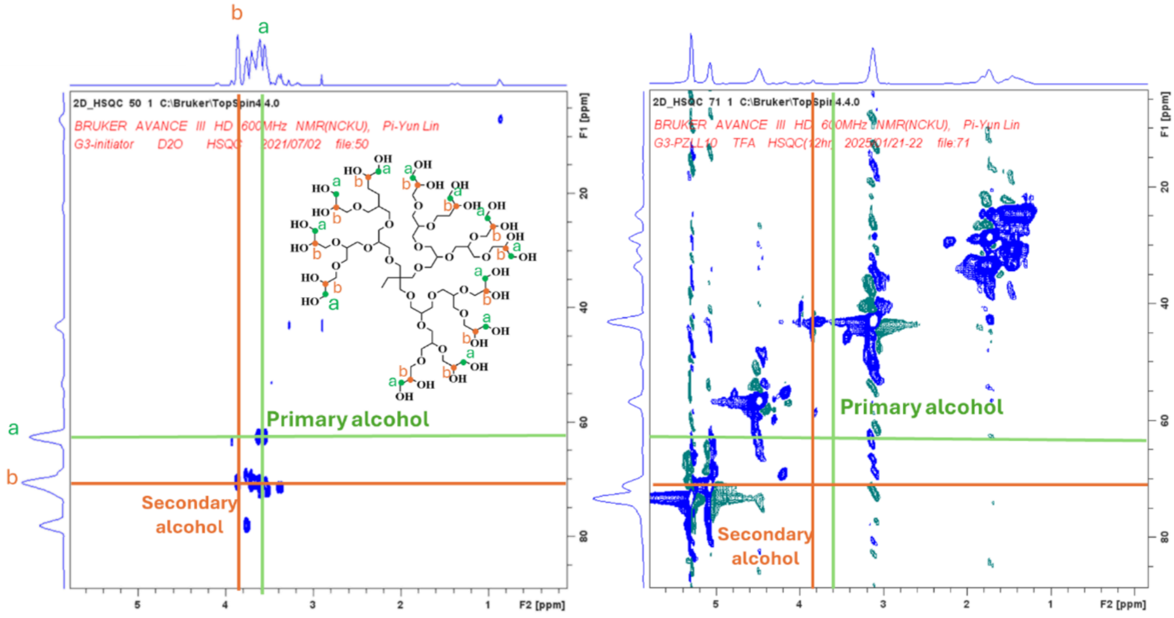

Figure S2. ^{1}H and ^{13}C NMR spectra showing (left) polyglycerol dendrimer of generation 3 (PGD-G3) initiator in D_2O and (right) G3-PZLL$_9$ in TFA-d_1.

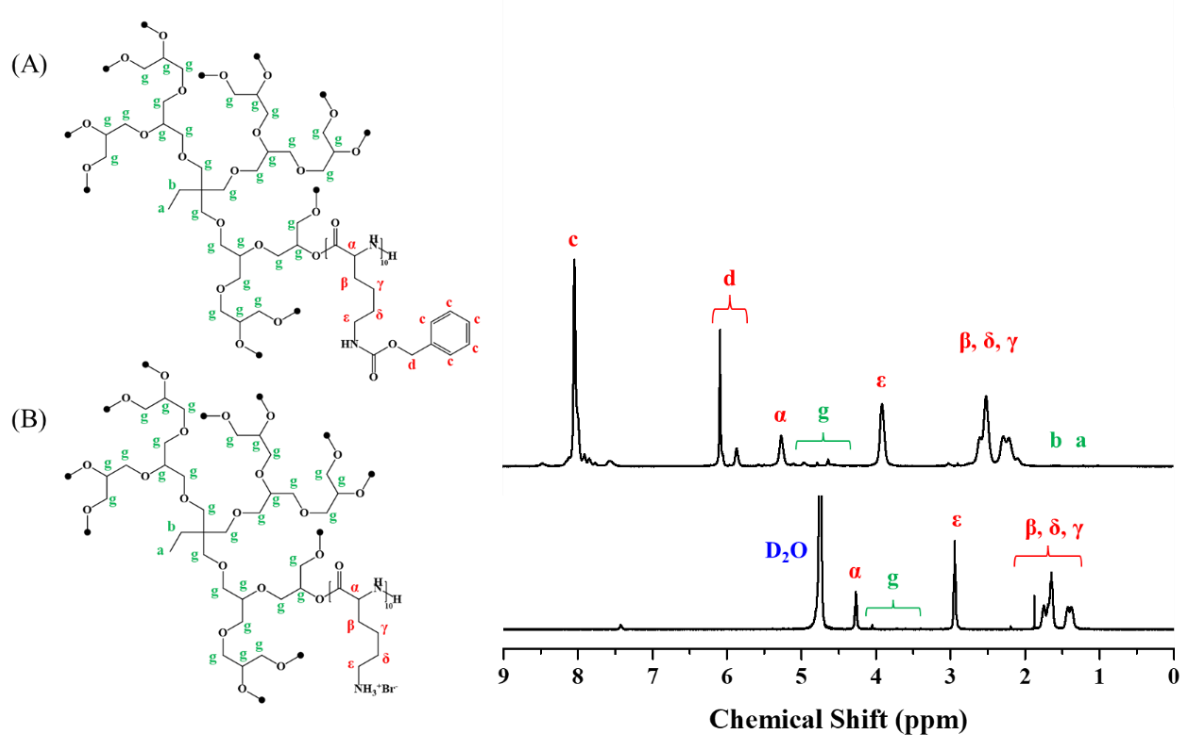

Figure S3. ^{1}H NMR spectra of (A) G2-PZLL$_{10}$ and (B) G2-PLL$_{10}$ dissolved in TFA-d_1 and D_2O, respectively.

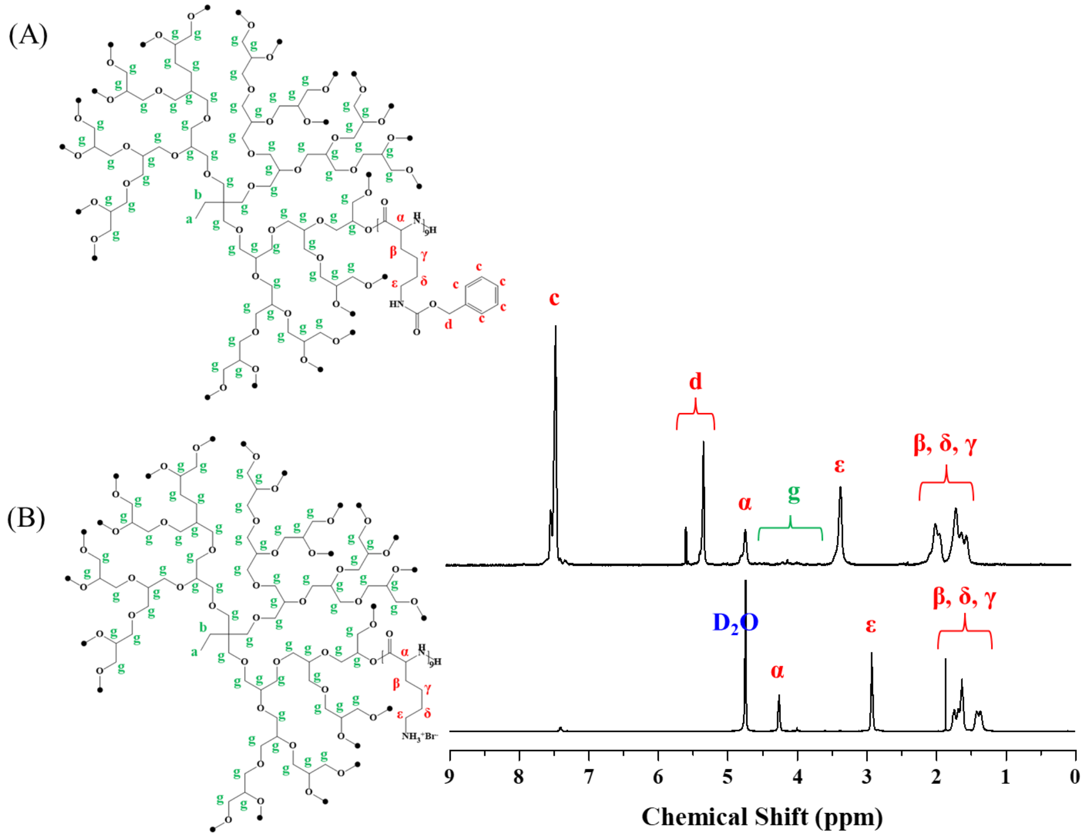

Figure S4. ^{1}H NMR spectra of (A) G3-PZLL9 and (B) G3-PLL9 dissolved in TFA-d_1 and D_2O, respectively.

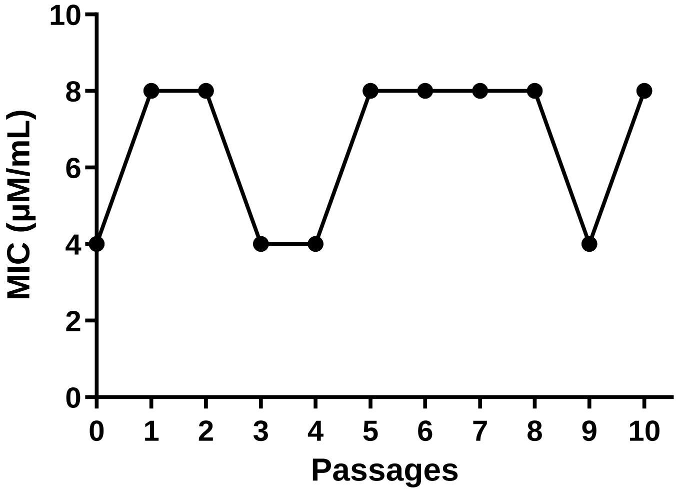

Figure S5. Serial passages of *C. difficile* (strain JIK 8284) after exposure to sub-MIC concentrations of G3-PLL_9.

Serial passage of *C. difficile* resulted in no more than a two-fold increase in MIC over ten consecutive passages, indicating a low potential for resistance development. MIC, minimum inhibitory concentration.

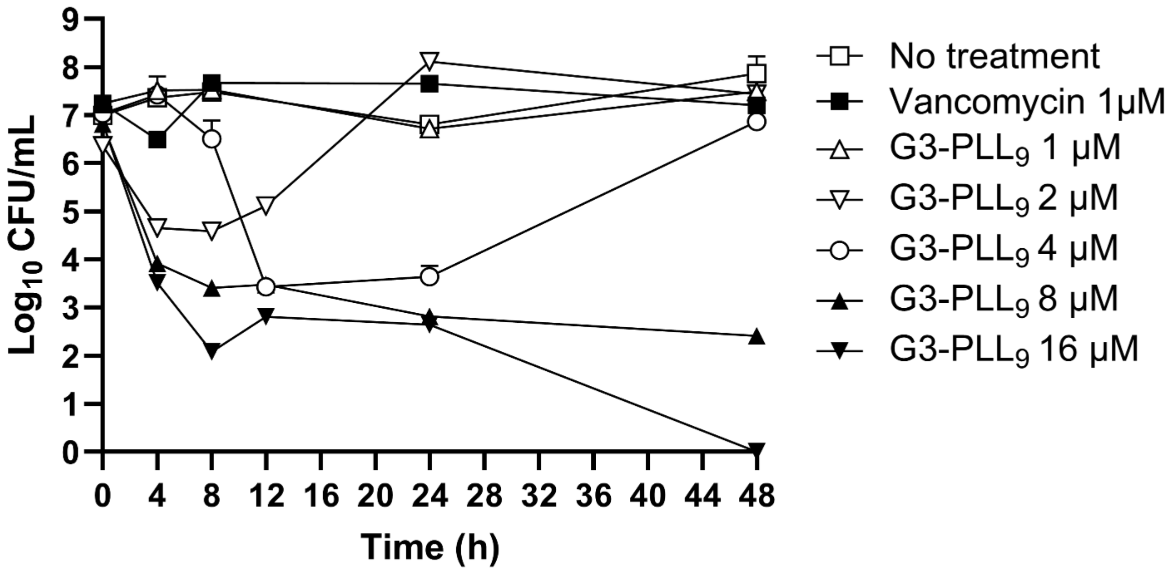

Figure S6. Time-kill growth curve analysis of G3-PLL9 against high-inoculum *C. difficile* (10^6–10^7 CFU mL^{-1}) in comparison to vancomycin at 1 μM. CFU, colony forming unit.

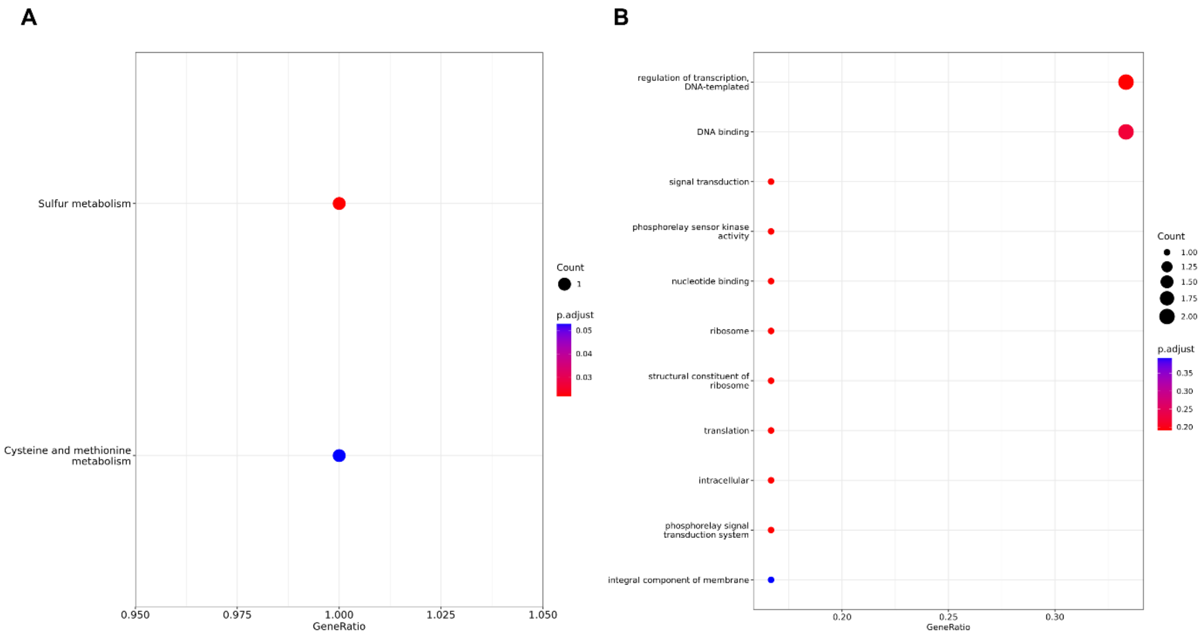

Figure S7. Kyoto Encyclopedia of Genes and Genomes (KEGG) and Gene Ontology (GO) enrichment analysis.

Enriched biological pathways based on KEGG (A) and GO databases (B). The plots show enriched terms on the y-axis and their corresponding GeneRatio (proportion of differentially expressed genes in the pathway relative to all genes in that pathway) on the x-axis. The dot size represents the number of genes in each pathway, and the color intensity indicates the adjusted *P*-value.

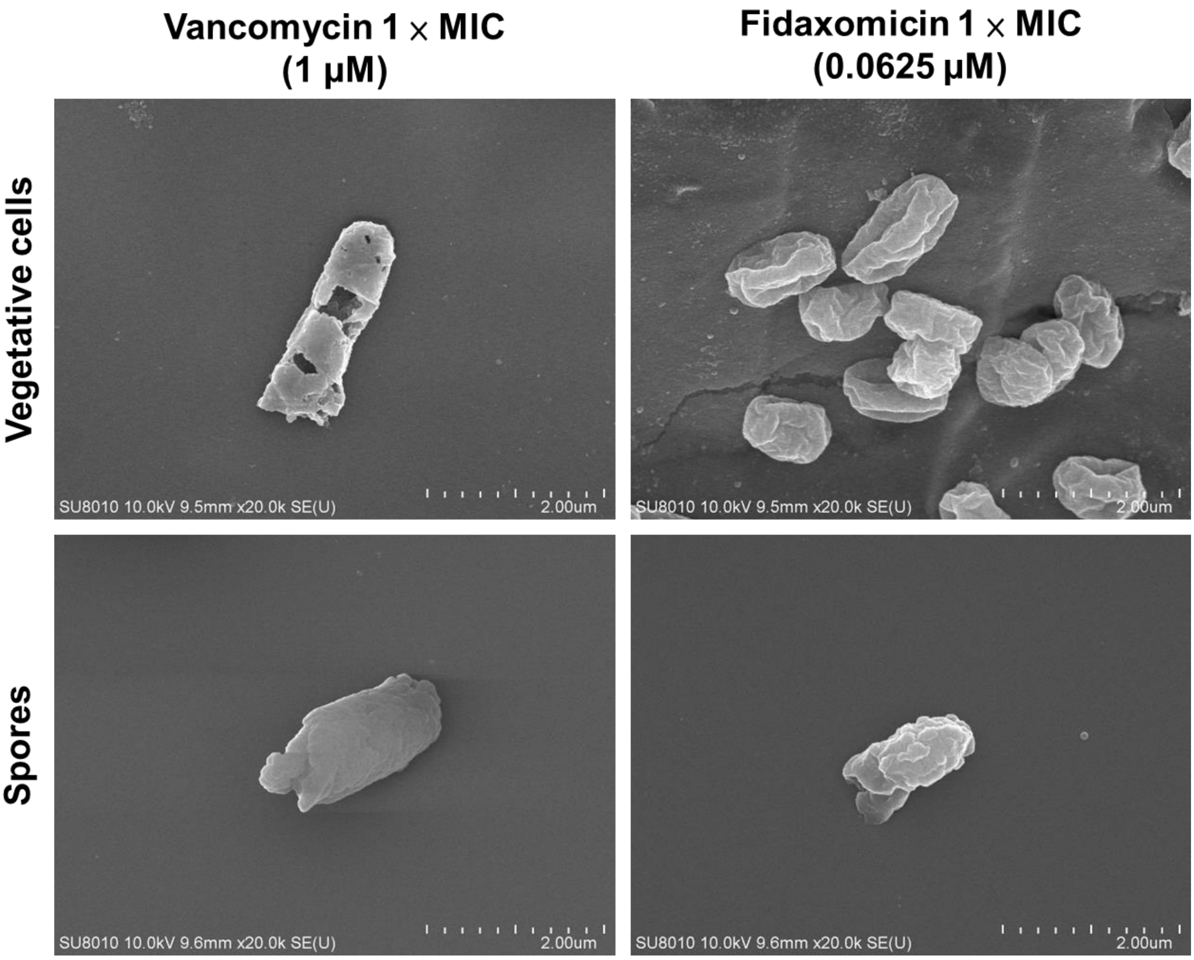

Figure S8. Scanning electron microscopy images of *C. difficile* vegetative cells and spores following treatment with vancomycin or fidaxomicin at their respective 1× minimum inhibitory concentrations (MICs).

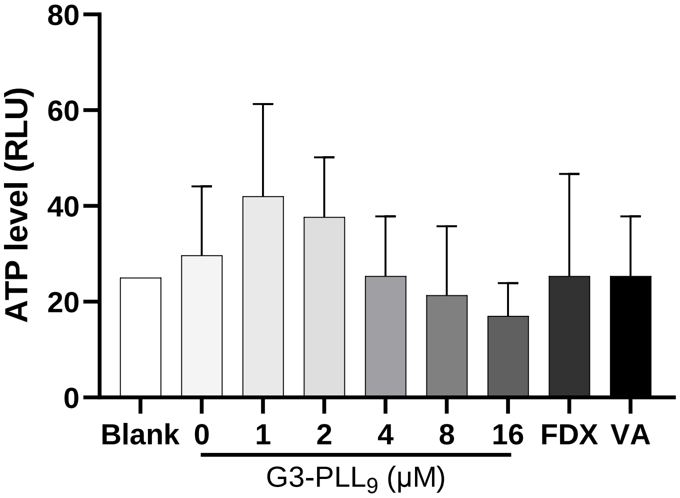

Figure S9. ATP-based viability of *C. difficile* following G3-PLL9 treatment.

Bacterial viability was assessed using the BacTiter-Glo™ assay. C. difficile strain JIK 8284 (1 × 10^4 cells per well) was treated with G3-PLL9 at concentrations of 0, 1, 2, 4, 8, or 16 μM, vancomycin (VA, 1× MIC), or fidaxomicin (FDX, 1× MIC) for 30 min. Blank controls contained medium without bacteria. Luminescence intensity is proportional to intracellular ATP levels and reflects viable bacterial counts. Data are presented as median ± interquartile range (n = 3; independent experiments). RLU, relative luminescence units.

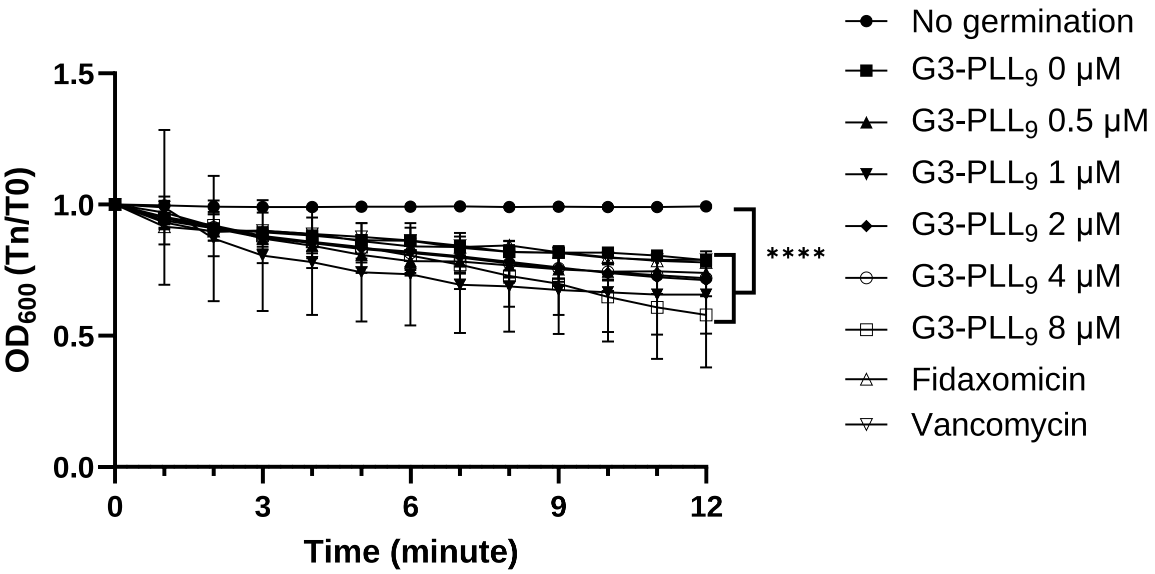

Figure S10. Germination inhibition assay of G3-PLL9 against *C. difficile* spores.

The graph shows the optical density (OD_{600}) change for *C. difficile* spore suspensions treated with various concentrations of G3-PLL9 or vancomycin at its minimum bactericidal concentration (1 μM) over 12 min. Data are presented as median ± interquartile range (n = 2, with biological repeats twice). A decrease in OD_{600} indicates spore germination. Statistical significance was evaluated using the Mann–Whitney test compared to the ungerminated group. $^{****}P < 0.0001$.

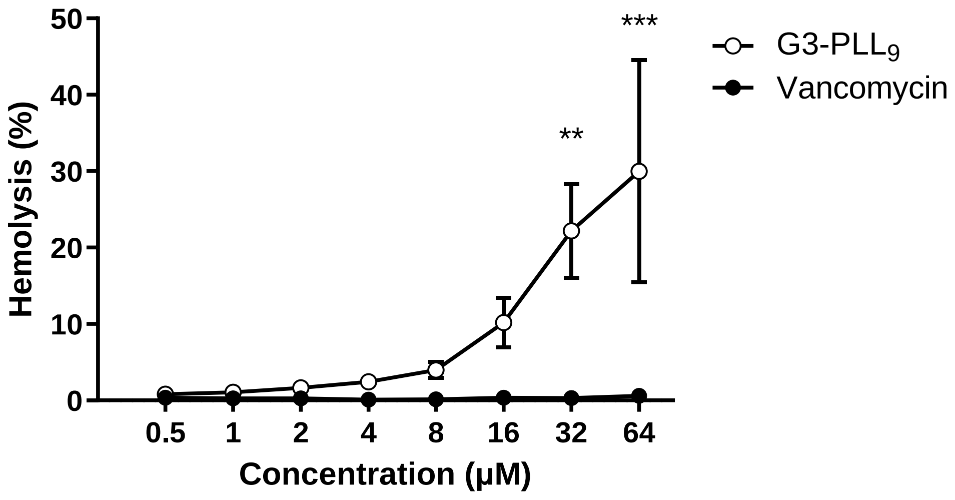

Figure S11. Hemolytic activity of G3-PLL$_9$ compared to vancomycin.

G3-PLL$_9$ exhibited no increase in hemolysis across the tested concentration range in this study (0.5–8 μM).

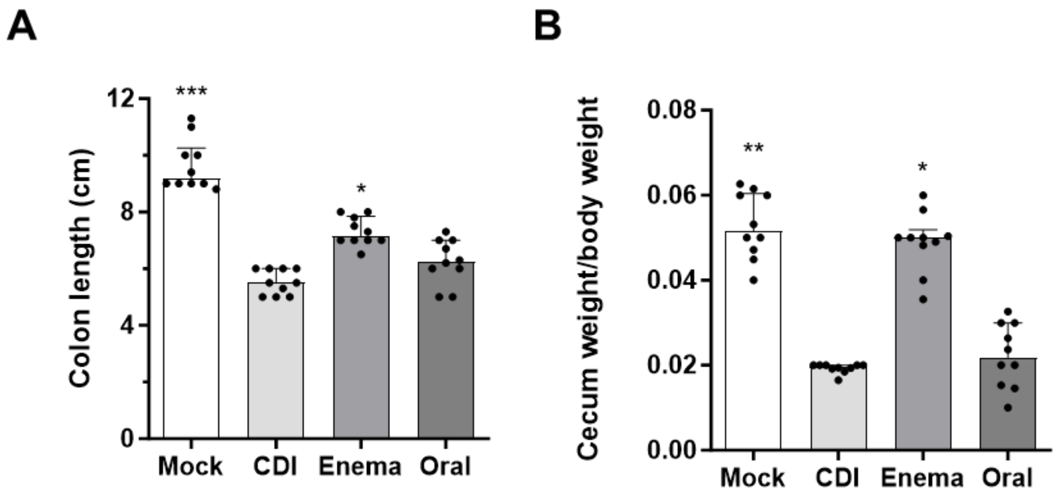

Figure S12. Efficacy of G3-PLL9 in *C. difficile* infection (CDI) treatment via oral versus enema administration.

(A–B) Therapeutic response indicators: colon length (A) and cecum weight (B) in a CDI mouse model. Data are presented as median ± interquartile range (n = 5 per group). Statistical comparisons between treatment groups and the CDI control were performed using the Kruskal–Wallis test with Dunn's post hoc test. *$P < 0.05$, **$P < 0.01$, ***$P < 0.001$.

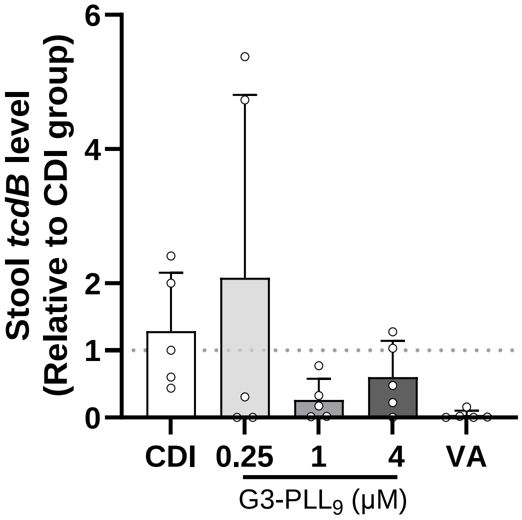

Figure S13. Quantification of stool *tcdB* levels using real-time polymerase chain reaction. Data are presented as median ± interquartile range (n = 5 in each group). CDI, *C. difficile* infection. VA, vancomycin.

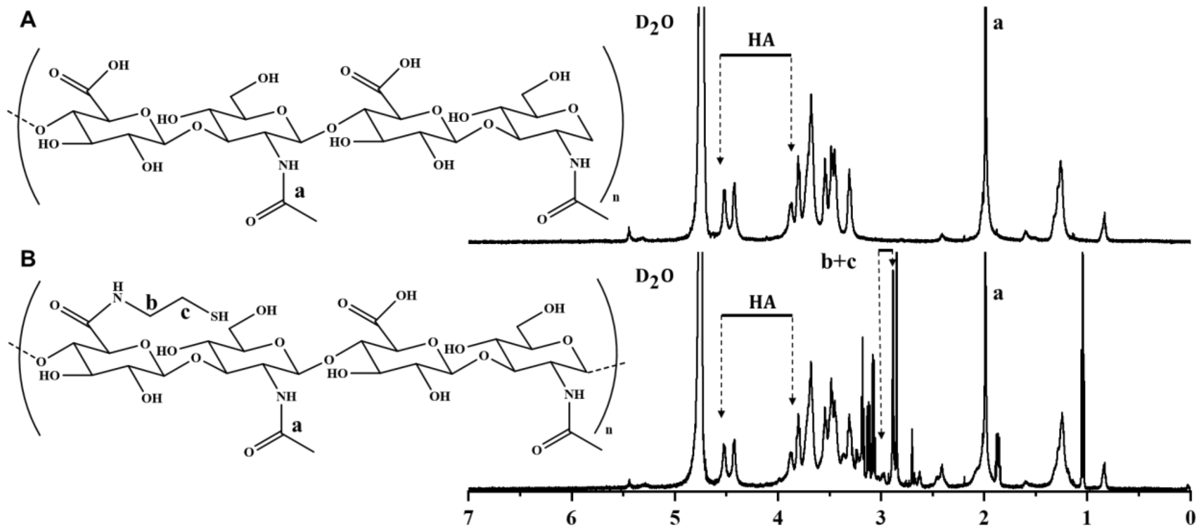

Figure S14. ^{1}H NMR spectra of (A) hyaluronic acid and (B) cysteamine-modified hyaluronic acid.

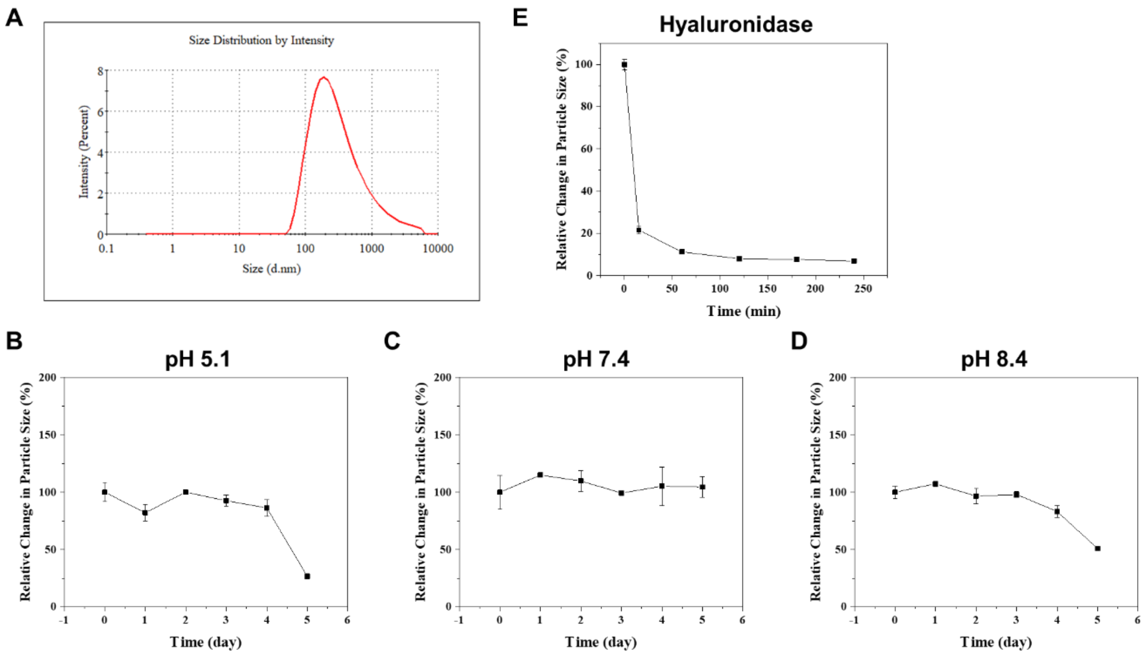

Figure S15. Characterization of HA/G3-PLL$_9$ microgels.

(A) HA/G3-PLL$_9$ particle size distribution plot.

(B–D) Particle size changes of HA/G3-PLL$_9$ microgels under various pH conditions, pH 5.1 (B), pH 7.4 (C), and pH 8.4 (D).

(E) Particle size changes of HA/G3-PLL$_9$ nanoparticles under hyaluronidase treatment (0.2 mg mL^{-1}).

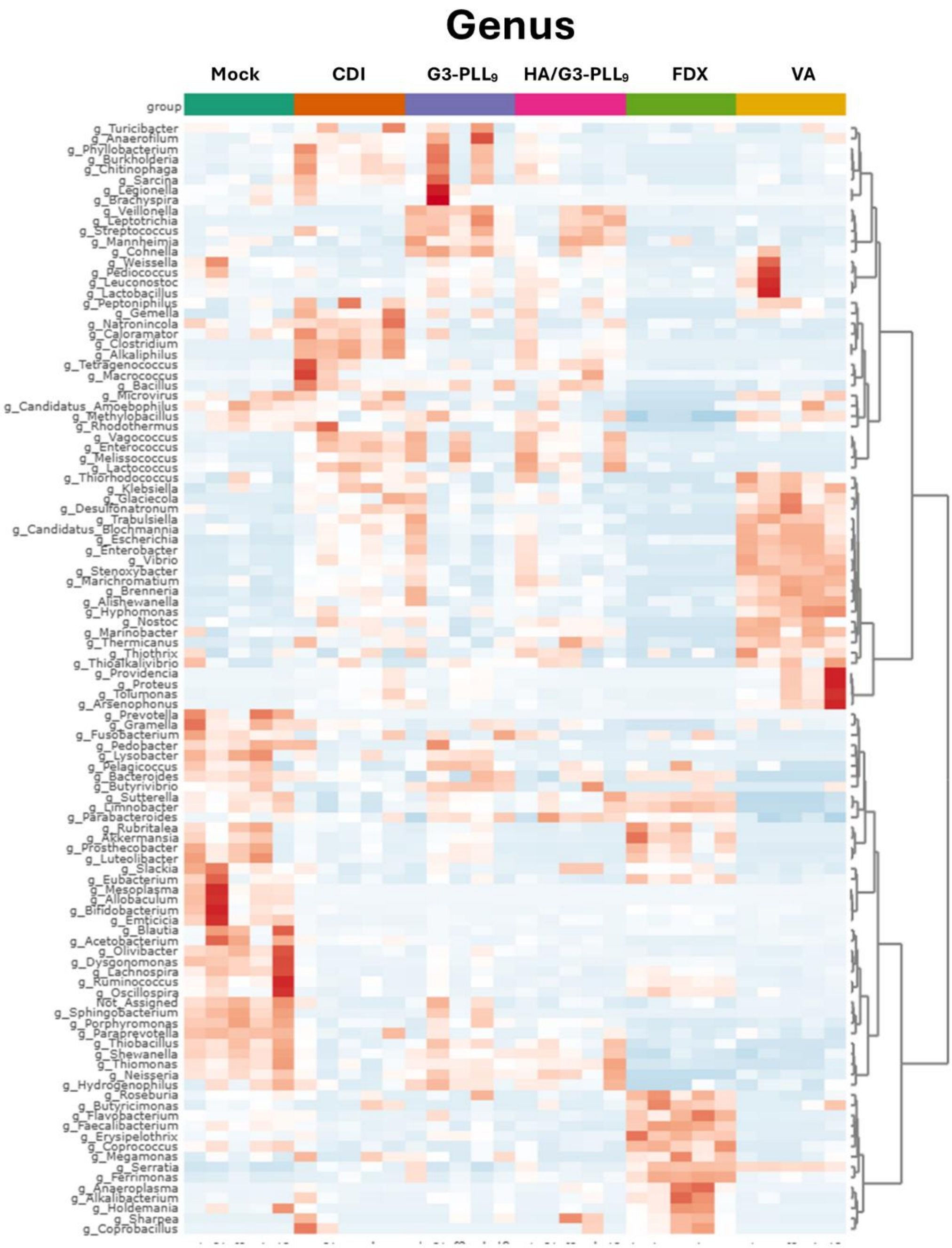
Genus
Mock
CDI
G3-PLL9
HA/G3-PLL9
FDX
VA
group
g_Turicibacter
g_Anaerofilum
g_Phyllobacterium
g_Burkholderia
g_Chitinophaga
g_Sarcina
g_Legionella
g_Brachyspira
g_Veillonella
g_Leptotrichia
g_Streptococcus
g_Mannheimia
g_Cohnella
g_Weissella
g_Pediococcus
g_Leuconostoc
g_Lactobacillus
g_Peptoniphilus
g_Gemella
g_Natronincola
g_Caloramator
g_Clostridium
g_Alkaliphilus
g_Tetragenococcus
g_Macrococcus
g_Bacillus
g_Microvirus
g_Candidatus_Amoebophilus
g_Methylobacillus
g_Rhodothermus
g_Vagococcus
g_Enterococcus
g_Melissococcus
g_Lactococcus
g_Thiorhodococcus
g_Klebsiella
g_Glaciecola
g_Desulfonatronum
g_Trabulsiella
g_Candidatus_Blochmannia
g_Escherichia
g_Enterobacter
g_Vibrio
g_Stenoxybacter
g_Marichromatium
g_Brenneria
g_Alishewanella
g_Hyphomonas
g_Nostoc
g_Marinobacter
g_Thermicanus
g_Thiothrix
g_Thioalkalivibrio
g_Providencia
g_Proteus
g_Tolumonas
g_Arsenophonus
g_Prevotella
g_Gramella
g_Fusobacterium
g_Pedobacter
g_Lysobacter
g_Pelagicoccus
g_Bacteroides
g_Butyrivibrio
g_Sutterella
g_Limnobacter
g_Parabacteroides
g_Rubritalea
g_Akkermansia
g_Prosthecobacter
g_Luteolibacter
g_Slackia
g_Eubacterium
g_Mesoplasma
g_Allobaculum
g_Bifidobacterium
g_Emticicia
g_Blautia
g_Acetobacterium
g_Olivibacter
g_Dysgonomonas
g_Lachnospira
g_Ruminococcus
g_Oscillospira
Not_Assigned
g_Sphingobacterium
g_Porphyromonas
g_Paraprevotella
g_Thiobacillus
g_Shewanella
g_Thiomonas
g_Neisseria
g_Hydrogenophilus
g_Roseburia
g_Butyricimonas
g_Flavobacterium
g_Faecalibacterium
g_Erysipelothrix
g_Coprococcus
g_Megamonas
g_Serratia
g_Ferrimonas
g_Anaeroplasma
g_Alkalibacterium
g_Holdemania
g_Sharpea
g_Coprobacillus

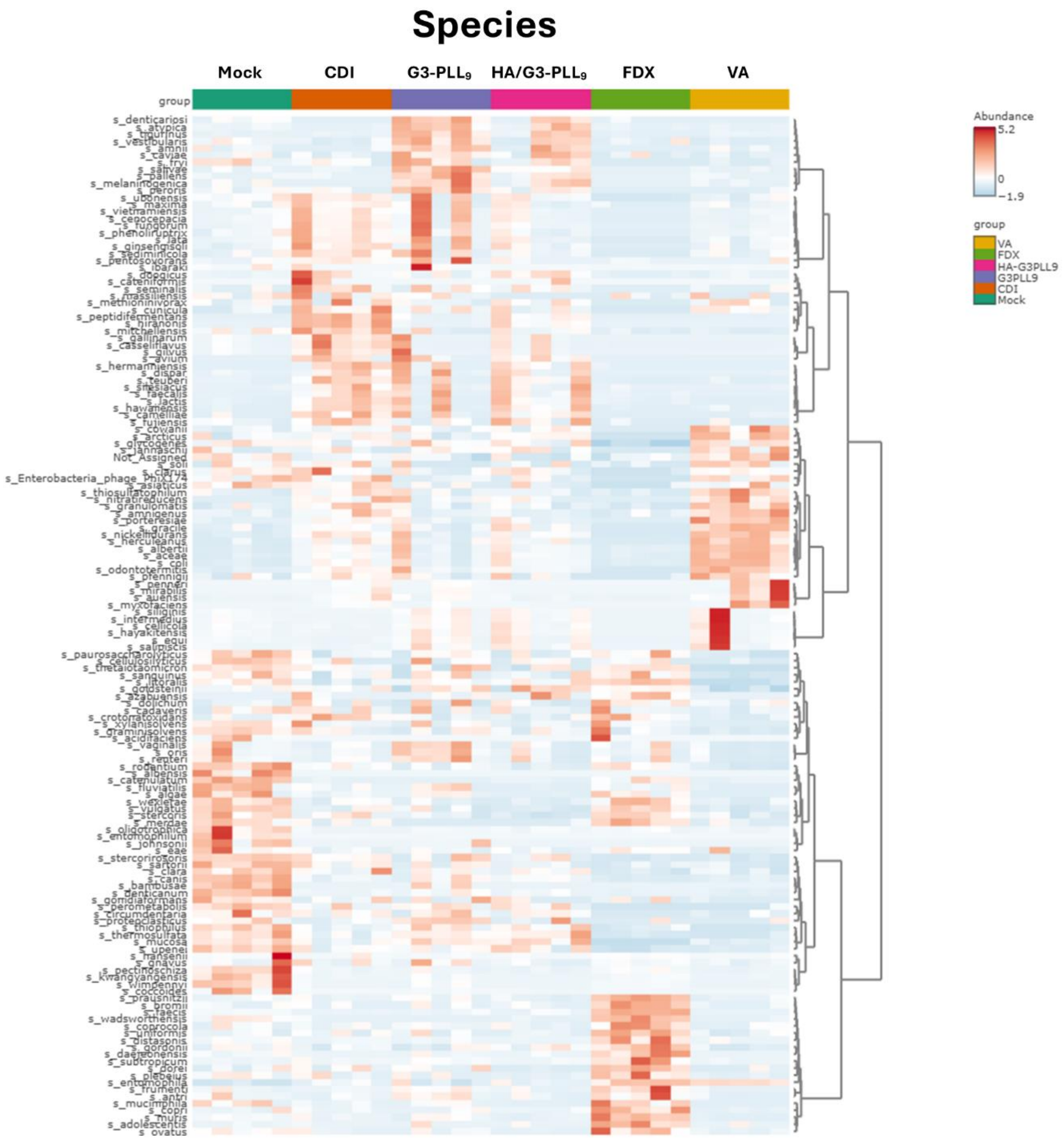

Figure S16. Microbiota composition after treatment for *C. difficile* infection (CDI). Fecal specimens were collected at the time of sacrifice. Hierarchical clustering heatmaps of gut microbiota composition at the genus (top) and species (bottom) levels. Color intensity represents relative abundance (red, high; blue, low).

References

(1) Edwards, A. N.; Suárez, J. M.; McBride, S. M. Culturing and Maintaining *Clostridium difficile* in an Anaerobic Environment. *J. Vis. Exp.* **2013**, (79), e50787. DOI: 10.3791/50787.

(2) Koeth, L. M. Minimum Bactericidal Concentration Testing. In *Clinical Microbiology Procedures Handbook*, 5th ed.; Leber, A. L., Ed.; ASM Press: Washington, DC, 2016; pp 5.14.1.1–5.14.3.6.

(3) Roth, S.; Jung, P.; Boone, J.; Mellmann, A.; Nimmesgern, A.; Becker, S. L.; Berger, F. K.; von Müller, L. Antigen-Specific vs. Neutralizing Antibodies Against Conditioned Media of Patients With *Clostridioides difficile* Infection: A Prospective Exploratory Study. *Front. Microbiol.* **2022**, *13*, 859037. DOI: 10.3389/fmicb.2022.859037.

(4) Edwards, A. N.; McBride, S. M. Isolating and Purifying *Clostridium difficile* Spores. *Methods Mol. Biol.* **2016**, *1476*, 117–128. DOI: 10.1007/978-1-4939-6361-4_9.

(5) Weldy, M.; Evert, C.; Dosa, P. I.; Khoruts, A.; Sadowsky, M. J. Convenient Protocol for Production and Purification of *Clostridioides difficile* Spores for Germination Studies. *STAR Protoc.* **2020**, *1* (2), 100071. DOI: 10.1016/j.xpro.2020.100071.

(6) Chen, Y. H.; Li, T. J.; Tsai, B. Y.; Chen, L. K.; Lai, Y. H.; Li, M. J.; Tsai, C. Y.; Tsai, P. J.; Shieh, D. B. Vancomycin-Loaded Nanoparticles Enhance Sporicidal and Antibacterial Efficacy for *Clostridium difficile* Infection. *Front. Microbiol.* **2019**, *10*, 1141. DOI: 10.3389/fmicb.2019.01141.

(7) Edwards, A. N.; Karim, S. T.; Pascual, R. A.; Jowhar, L. M.; Anderson, S. E.; McBride, S. M. Chemical and Stress Resistances of *Clostridium difficile* Spores and Vegetative Cells. *Front. Microbiol.* **2016**, *7*, 1698. DOI: 10.3389/fmicb.2016.01698.

(8) Rodriguez-Palacios, A.; LeJeune, J. T. Moist-Heat Resistance, Spore Aging, and Superdormancy in *Clostridium difficile*. *Appl. Environ. Microbiol.* **2011**, *77* (9), 3085–3091. DOI: 10.1128/AEM.01589-10.

(9) Pickering, D. S.; Vernon, J. J.; Freeman, J.; Wilcox, M. H.; Chilton, C. H. Investigating the Transient and Persistent Effects of Heat on *Clostridium difficile* Spores. *J. Med. Microbiol.* **2019**, *68* (10), 1445–1454. DOI: 10.1099/jmm.0.001048.

(10) Sorg, J. A.; Sonenshein, A. L. Chenodeoxycholate Is an Inhibitor of *Clostridium difficile* Spore Germination. *J. Bacteriol.* **2009**, *191* (3), 1115–1117. DOI: 10.1128/jb.01260-08.

(11) Piotrowski, M.; Karpiński, P.; Pituch, H.; van Belkum, A.; Obuch-Woszczatyński, P. Antimicrobial Effects of Manuka Honey on In Vitro Biofilm Formation by *Clostridium difficile*. *Eur. J. Clin. Microbiol. Infect. Dis.* **2017**, *36* (9), 1661–1664. DOI: 10.1007/s10096-017-2980-1.

(12) Lu, Y.; Aizhan, R.; Yan, H.; Li, X.; Wang, X.; Yi, Y.; Shan, Y.; Liu, B.; Zhou, Y.; Lü, X. Characterization, Modes of Action, and Application of a Novel Broad-Spectrum Bacteriocin

BM1300 Produced by *Lactobacillus crustorum* MN047. *Braz. J. Microbiol.* **2020**, *51* (4), 2033–2048. DOI: 10.1007/s42770-020-00311-3.

(13) Hung, Y. P.; Ko, W. C.; Chou, P. H.; Chen, Y. H.; Lin, H. J.; Liu, Y. H.; Tsai, H. W.; Lee, J. C.; Tsai, P. J. Proton-Pump Inhibitor Exposure Aggravates *Clostridium difficile*-Associated Colitis: Evidence from a Mouse Model. *J. Infect. Dis.* **2015**, *212* (4), 654–663. DOI: 10.1093/infdis/jiv184.

(14) Erikstrup, L. T.; Aarup, M.; Hagemann-Madsen, R.; Dagnaes-Hansen, F.; Kristensen, B.; Olsen, K. E.; Fuursted, K. Treatment of *Clostridium difficile* Infection in Mice with Vancomycin Alone Is as Effective as Treatment with Vancomycin and Metronidazole in Combination. *BMJ Open Gastroenterol.* **2015**, *2* (1), e000038. DOI: 10.1136/bmjgast-2015-000038.

(15) Malamood, M.; Nellis, E.; Ehrlich, A. C.; Friedenberg, F. K. Vancomycin Enemas as Adjunctive Therapy for *Clostridium difficile* Infection. *J. Clin. Med. Res.* **2015**, *7* (6), 422–427. DOI: 10.14740/jocmr2117w.

(16) Fawley, J.; Napolitano, L. M. Vancomycin Enema in the Treatment of *Clostridium difficile* Infection. *Surg. Infect.* **2019**, *20* (4), 311–316. DOI: 10.1089/sur.2018.238.

(17) Houser, B. A.; Hattel, A. L.; Jayarao, B. M. Real-Time Multiplex Polymerase Chain Reaction Assay for Rapid Detection of *Clostridium difficile* Toxin-Encoding Strains. *Foodborne Pathog. Dis.* **2010**, *7* (6), 719–726. DOI: 10.1089/fpd.2009.0483.

(18) Bacchetti De Gregoris, T.; Aldred, N.; Clare, A. S.; Burgess, J. G. Improvement of Phylum- and Class-Specific Primers for Real-Time PCR Quantification of Bacterial Taxa. *J. Microbiol. Methods* **2011**, *86* (3), 351–356. DOI: 10.1016/j.mimet.2011.06.010.

(19) Livak, K. J.; Schmittgen, T. D. Analysis of Relative Gene Expression Data Using Real-Time Quantitative PCR and the 2^−ΔΔCT^ Method. *Methods* **2001**, *25* (4), 402–408. DOI: 10.1006/meth.2001.1262.

(20) Lin, J. H.; Lin, C. H.; Kuo, Y. W.; Liao, C. A.; Chen, J. F.; Tsai, S. Y.; Li, C. M.; Hsu, Y. C.; Huang, Y. Y.; Hsia, K. C.; Shieh, M. J.; Sheu, M. J.; Hou, C. Y. Probiotic *Lactobacillus fermentum* TSF331, *Lactobacillus reuteri* TSR332, and *Lactobacillus plantarum* TSP05 Improved Liver Function and Uric Acid Management—A Pilot Study. *PLoS One* **2024**, *19* (7), e0307181. DOI: 10.1371/journal.pone.0307181.

(21) Tsai, W.-H.; Yeh, W.-L.; Chou, C.-H.; Wu, C.-L.; Lai, C.-H.; Yeh, Y.-T.; Liao, C.-A.; Wu, C.-C. Suppressive Effects of *Lactobacillus* on Depression through Regulating the Gut Microbiota and Metabolites in C57BL/6J Mice Induced by Ampicillin. *Biomedicines* **2023**, *11* (4), 1068.

(22) Shelby, R. D.; Tengberg, N.; Conces, M.; Olson, J. K.; Navarro, J. B.; Bailey, M. T.; Goodman, S. D.; Besner, G. E. Development of a Standardized Scoring System to Assess a Murine Model of *Clostridium difficile* Colitis. *J. Invest. Surg.* **2020**, *33* (10), 887–895. DOI: 10.1080/08941939.2019.1571129.
